# Role of cardiolipins, mitochondria, and autophagy in the differentiation process activated by all-trans retinoic acid in acute promyelocytic leukemia

**DOI:** 10.1038/s41419-021-04476-z

**Published:** 2022-01-10

**Authors:** Maurizio Gianni’, Laura Goracci, Anna Schlaefli, Alessandra Di Veroli, Mami Kurosaki, Luca Guarrera, Marco Bolis, Marika Foglia, Monica Lupi, Mario P. Tschan, Gabriele Cruciani, Mineko Terao, Enrico Garattini

**Affiliations:** 1 Laboratory of Molecular Biology, Istituto di Ricerche Farmacologiche Mario Negri IRCCS, via Mario Negri 2, 20156 Milano, Italy; 2 Department of Chemistry, Biology and Biotechnology, University of Perugia, via Elce di Sotto 8, 06123 Perugia, Italy; 3 Institute of Pathology, University of Bern, Murtenstrasse 31, CH-3008 Bern, Switzerland; 4 Functional Cancer Genomics Laboratory, Institute of Oncology Research, USI, University of Southern Switzerland, 6500 Bellinzona, Switzerland; 5 Bioinformatics Core Unit Institute of Oncology Research, Swiss Institute of Bioinformatics, 1000 Lausanne, Switzerland; 6 Department of Oncology, Istituto di Ricerche Farmacologiche “Mario Negri” IRCCS, via Mario Negri 2, 20156 Milano, Italy

**Keywords:** Cancer, Cell biology

## Abstract

The role played by lipids in the process of granulocytic differentiation activated by all-trans retinoic acid (ATRA) in Acute-Promyelocytic-Leukemia (APL) blasts is unknown. The process of granulocytic differentiation activated by ATRA in APL blasts is recapitulated in the *NB4* cell-line, which is characterized by expression of the pathogenic PML-RARα fusion protein. In the present study, we used the *NB4* model to define the effects exerted by ATRA on lipid homeostasis. Using a high-throughput lipidomic approach, we demonstrate that exposure of the APL-derived *NB4* cell-line to ATRA causes an early reduction in the amounts of cardiolipins, a major lipid component of the mitochondrial membranes. The decrease in the levels of cardiolipins results in a concomitant inhibition of mitochondrial activity. These ATRA-dependent effects are causally involved in the granulocytic maturation process. In fact, the ATRA-induced decrease of cardiolipins and the concomitant dysfunction of mitochondria precede the differentiation of retinoid-sensitive *NB4* cells and the two phenomena are not observed in the retinoid-resistant *NB4.306* counterparts. In addition, ethanolamine induced rescue of the mitochondrial dysfunction activated by cardiolipin deficiency inhibits ATRA-dependent granulocytic differentiation and induction of the associated autophagic process. The *RNA-seq* studies performed in parental *NB4* cells and a *NB4*-derived cell population, characterized by silencing of the autophagy mediator, ATG5, provide insights into the mechanisms underlying the differentiating action of ATRA. The results indicate that ATRA causes a significant down-regulation of CRLS1 (Cardiolipin-synthase-1) and LPCAT1 (Lysophosphatidylcholine-Acyltransferase-1) mRNAs which code for two enzymes catalyzing the last steps of cardiolipin synthesis. ATRA-dependent down-regulation of CRLS1 and LPCAT1 mRNAs is functionally relevant, as it is accompanied by a significant decrease in the amounts of the corresponding proteins. Furthermore, the decrease in CRLS1 and LPCAT1 levels requires activation of the autophagic process, as down-regulation of the two proteins is blocked in ATG5-silenced *NB4-shATG5* cells.

## Introduction

All-trans retinoic acid (ATRA) is a differentiating agent used in the treatment of Acute-Promyelocytic-Leukemia (APL), a rare form of Acute-Myeloid-Leukemia (AML) [1–6]. The blasts of over 90% of APL patients show a specific t(15;17) chromosomal translocation which leads to the expression of the oncogenic PML-RARα fusion protein [7] and contains RARα, the main retinoid receptor expressed in hematopoietic cells. The differentiation process induced by ATRA is recapitulated in cultures of the APL-derived and PML-RARα expressing *NB4* cell-line [8, 9].

The role played by lipids in the process of granulocytic maturation activated by ATRA in APL blasts is unknown. Here, we use the *NB4* cellular model [10–12] to establish that ATRA causes an early decrease of cardiolipins (*CLs*), a major lipid component of the mitochondria. Functional and transcriptomic studies provide insights into the cellular/molecular events activated by the decrease in *CLs* levels, which are involved in the APL-blast differentiation process activated by ATRA.

## Results

### ATRA-dependent perturbations of the lipidomic profiles in NB4 cells

To define the composition of cellular lipids during the differentiation process activated by ATRA in APL blasts, we used retinoid-sensitive *NB4* and retinoid-resistant *NB4.306* cells [13–17]. *NB4* and *NB4.306* cells were exposed to vehicle (DMSO) or ATRA (1 μM) for 6, 24, and 48 h, three time-points preceding the retinoid-dependent differentiation of *NB4* cells into granulocytes which starts to be evident following 72–96 h [9]. Using a lipidomic approach [18], we determined the number and types of lipids observed in the two cell-lines. Principal-component-analysis (PCA) indicates that *NB4* and *NB4.306* cells present with similar lipidomic profiles under basal conditions and the perturbations induced by ATRA are limited (Supplementary Fig. S1A). Indeed, we identify 339 entities belonging to 12 different lipid sub-classes. Each sub-class consists of a variable number of entities ranging from 1 to 121 (Supplementary Fig. S1B and Supplementary Table. S1).

**Fig. 1:**
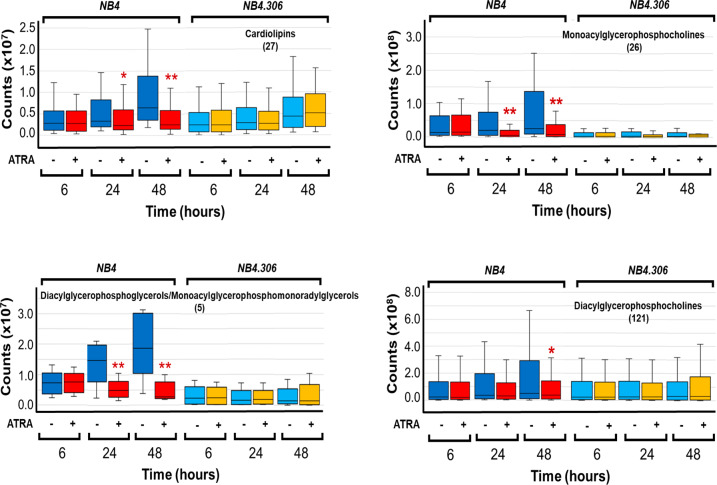
ATRA effects on the levels of selected lipid species in NB4 and NB4.306 cells. Biological triplicates of the indicated retinoid-sensitive *NB4* and retinoid-resistant *NB4.306* cells were treated with vehicle (DMSO) or ATRA (1 μM) for the indicated amounts of time. The box plots show the median ± SD levels of the indicated lipid species. The number of different molecules identified by mass-spectrometry is indicated in parenthesis. *Significantly different from the corresponding vehicle treated control (*p* < 0.05, unpaired Student’s *t* test). **Significantly different (*p* < 0.01) from the corresponding vehicle treated control (*p* < 0.01, unpaired Student’s *t* test).

In both *NB4* and *NB4.306* cells, ATRA exerts no significant effect on the overall levels of various lipid sub-classes (Supplementary Table. S1 and Supplementary Fig. S2). In *NB4* cells (Fig.1), ATRA causes a selective down-regulation of *CLs* (27 entities), *monoacylglycerophosphocholines* (26 entities) and *diacylglycerophosphoglycerols/monoacylglycerophosphomonoradylglycerols* (5 entities). The reduction in the amounts of these lipids starts at 24 h and it is maintained at 48 h. The sole lipid sub-class showing an ATRA-dependent decrease only at 48 h is represented by *diacylglycerophosphocholines* (121 entities). In *NB4.306* cells, ATRA does not alter the composition and levels of the 4 lipid groups. Thus, down-regulation of *CLs*, *monoacylglycerophosphocholines*,*diacylglycerophosphoglycerols/Monoacylglycerophosphomonoradylglycerols* and *diacylglycerophosphocholines* are associated with sensitivity to the ATRA-differentiating activity. These four lipid-types are glycerophospholipids belonging to the same metabolic pathway (KEGG PATHWAY: map00564, glycerophospholipid metabolism). Hence, ATRA-induced differentiation of *NB4* cells may alter the biosynthesis/degradation of membrane glycerophospholipids [19].

### ATRA-dependent effects on the activity of mitochondria in NB4 and NB4.306 cells

We focussed on *CLs*, as these lipids are reduced by ATRA also in breast-cancer cell-lines [18]. *CLs* localize to mitochondrial membranes and are involved in the bio-energetic processes [20, 21]. Thus, we evaluated whether *CLs* down-regulation in retinoid-sensitive *NB4* cells is associated with alterations in mitochondrial homeostasis. We exposed *NB4* and *NB4.306* blasts to ATRA for 24–72 h and co-stained the cells with Mito-Tracker green and Mito-Tracker red before conducting FACS analyses. The levels of Mito-Tracker green-fluorescence provide an estimate of the total mass of mitochondria. In contrast, the levels of Mito-Tracker red/green-fluorescence (red-fluorescence following normalization for green-fluorescence) define the functional activity of mitochondria (the higher is the ratio, the higher is mitochondrial activity) [22].

**Fig. 2:**
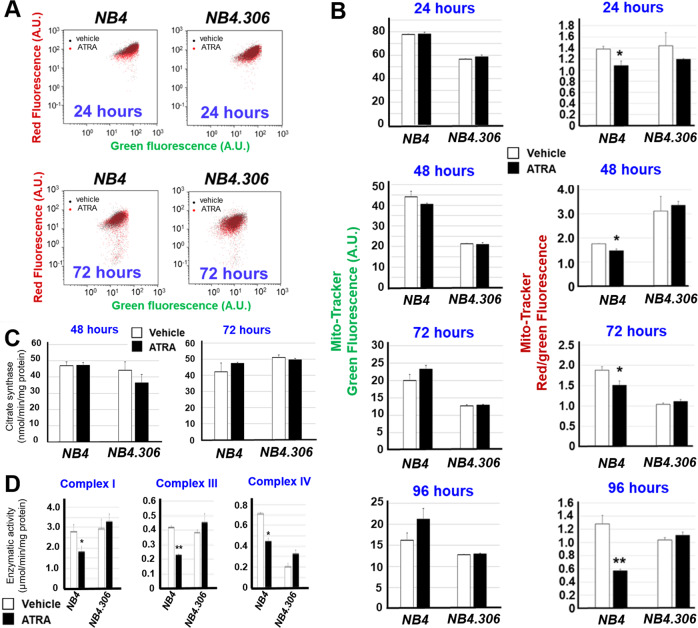
ATRA reduces the activity of mitochondria in sensitive NB4 cells. Three independent cultures of retinoid-sensitive *NB4* and retinoid-resistant *NB4.306* cells were treated with vehicle (DMSO) or ATRA (1 μM) for 24, 48, 72 and 96 h. At the end of the treatment, cells were contemporaneously stained with Mito-Tracker red and Mito-Tracker green fluorescent dyes and subjected to FACS analysis. **A** The panel shows representative FACS plots obtained following 24 and 72 h of exposure to ATRA. As indicated, the black dots refer to cells treated with vehicle (DMSO), while the red dots refers to cells treated with ATRA. **B** The column diagrams illustrate the quantification of the data obtained by FACS analysis of the three cultures of *NB4* and *NB4.306* cells. The left diagrams show the levels of Mito-Tracker green fluorescence. The right diagrams show the values of Mito-Tracker red/green fluorescence (red fluorescence following normalization for the green fluorescence value). Each value is the mean + S.D. of three samples. *Significantly different relative to the corresponding vehicle treated controls (*p* < 0.05, unpaired Student’s *t* test) **Significantly different relative to the corresponding vehicle treated controls (*p* < 0.01, unpaired Student’s *t* test). The results shown are representative of three independent experiments producing similar results. **C** Three cultures of *NB4* and *NB4.306* cells were treated with vehicle (DMSO) or ATRA (1 μM) for 48 and 72 h. At the end of the treatment total cell homogenates were used to determine citrate synthase activity. Each value is the mean + S.D. of three samples run in duplicate (*N* = 6). The data are representative of two independent experiments producing similar results. **D** Mitochondria were isolated from *NB4* and *NB4.306* cells cultured in the presence of vehicle or ATRA (1 μM) for 48 h. Mitochondrial Complex I, Complex III, and Complex IV enzymatic activities were measured on five replicates and the results are expressed as the Mean + SD. The data are representative of two independent experiments providing similar results.

ATRA exerts no significant effect on Mito-Tracker green-fluorescence in either *NB4* or *NB4.306* cells at any of the time points considered (Fig. 2A, B), which indicates that the retinoid does not alter the mitochondrial mass. Consistent with this, exposure of *NB4* or *NB4.306* cells to ATRA for 48 and 72 h does not alter the activity of citrate synthase, which is a quantitative marker of intact mitochondria (Fig. 2C). By converse, ATRA reduces Mito-Tracker red/green-fluorescence in retinoid-sensitive *NB4* cells at all time-points (Fig. 2A, B). A similar effect is not observed in the retinoid-resistant *NB4.306* counterparts. In *NB4* cells, the ATRA-induced decrease in Mito-Tracker red/green-fluorescence is long-lasting and it is maintained at least until 96 h. This indicates that ATRA-dependent down-regulation of *CLs* causes a reduction in the activity of mitochondria. The contention that ATRA reduces only the functional activity of mitochondria is further supported by the effects on Complex-I, Complex-III and Complex-IV enzymatic activities, which are diminished upon exposure of *NB4* cells to the retinoid for 48 h (Fig. 2D). Significantly, ATRA does not alter the activity of the three complexes in *NB4.306* cells.

### Ethanolamine effects on ATRA-dependent perturbations of mitochondrial-homeostasis, granulocytic-differentiation and autophagy

To evaluate whether the decrease in *CLs* levels and mitochondrial activity is relevant for the differentiation process activated by ATRA in *NB4* cells, we performed studies using an ethanolamine supplementation strategy [23]. In fact, ethanolamine rescues the mitochondrial insufficiency observed in *CLs* deficient yeast cells [24] *via* an increase in mitochondrial phosphatidylethanolamine [25], which is involved in mature *CLs* remodelling [21].

**Fig. 3:**
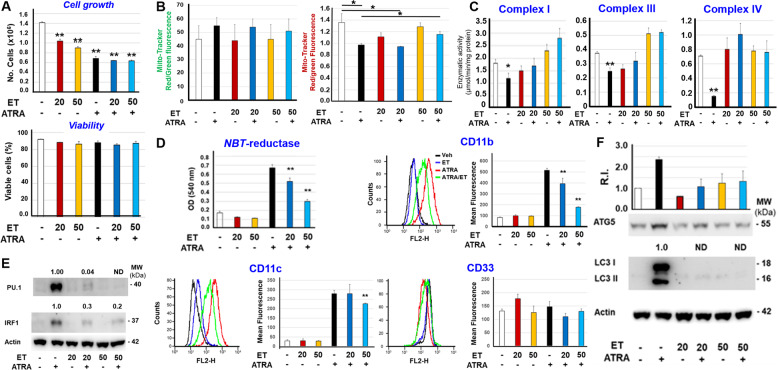
Effects of ethanolamine on the growth, viability, mitochondrial activity, differentiation, and autophagy of NB4 cells. Three independent cultures of retinoid-sensitive *NB4* cells were pre-treated with vehicle (DMSO), 20 and 50 μM ethanolamine (ET) for 12 h. Subsequently cells were treated with vehicle or ATRA (1 μM) for another 48 h. The concentrations of ET used (20 μM; 50 μM) are indicated by the numbers shown under the diagram. **A** Upper diagram: The number of cells was evaluated with the use of a Cell Viable Analyzer. Each value is the Mean + SD of three replicate and independent cultures. **Significantly different relative to the vehicle pretreated and vehicle treated cells (white column; *p* < 0.01, unpaired Student’s *t* test). Lower diagram: the viability of cells was evaluated with the use of a Cell Viable Analyzer and the results are expressed as the percentage of viable cells. Each value is the Mean + SD of three replicate and independent cultures. The diagrams shown are representative of two independent experiments producing similar results. **B** At the end of the treatment, cells were co-stained with Mito-Tracker green and Mito-Tracker red before subjecting them to FACS analysis. The left diagrams show the levels of Mito-Tracker green fluorescence. The right diagrams show the values of MitoTracker-red/green fluorescence (red fluorescence following normalization for the green fluorescence value). Each value is the Mean + SD of the three independent cultures. *Significantly different (*p* < 0.05, unpaired Student’s *t* test). The diagrams shown are representative of two independent experiments producing similar results. **C** Mitochondria were isolated from *NB4* cells exposed to vehicle, ET, ATRA or the combination of the two compounds as indicated. Mitochondrial Complex I, Complex III, and Complex IV enzymatic activities were measured on four replicates and the results are expressed as the Mean + SD. The data are representative of two independent experiments providing similar results. **Significantly lower relative to the corresponding control sample pre-treated and exposed to vehicle alone (white column; *p* < 0.01, unpaired Student’s *t* test). *Significantly lower relative to the corresponding control sample pre-treated and exposed to vehicle alone (white column; *p* < 0.05, unpaired Student’s *t* test). **D** The panel shows the expression levels of the myeloid markers, *NBT*-reductase, CD11b, CD11c, and CD33 as indicated. *NBT*-reductase activity was determined with a spectrophotometric assay. The surface expression of CD11b, CD11c and CD33 was determined by FACS analysis. In the case of CD11b, CD11c and CD33, the leftmost diagrams show representative FACS curves. The rightmost column diagrams illustrate the quantitative data obtained on the three independent samples analyzed. The results are expressed as the Mean + S.D. (*N* = 3) of the values determined. **Significantly lower relative to the corresponding control sample treated with ATRA alone (black column) (*p* < 0.01, unpaired Student’s *t* test). The diagrams shown are representative of two independent experiments producing similar results. **E** and **F** The panels illustrate the results of Western blot experiments performed on pooled cellular extracts obtained from three separate cultures of *NB4* cells exposed to vehicle, ET, ATRA or the combination of the two compounds, as indicated. The antibodies used target the differentiation markers, PU.1 and IRF1 (**E**), as well as the autophagic markers ATG5 and LC3I/LC3II (**F**). The same amounts of proteins were loaded in each lane, as demonstrated by the Western blot signals obtained with the anti-β_2_actin antibodies. In the case of PU.1, IRF1 and LC3I-II, the Western blots shown are representative of two independent experiments producing similar results. The numbers above the PU.1 LC3I-II and IRF1 lanes show the densitometric data of the Western blot. The values represent the PU.1/Actin, IRF1/Actin or LC3I-II/Actin ratios and are normalized for the results obtained after treatment with ATRA (lane 2 from the left). The PU.1/Actin or IRF1/Actin values are taken as 1.0. In the case of ATG5, the Western blots shown are representative of three experiments. The diagrams above the ATG5 lanes show the densitometric data of the Western blots performed in the three independent experiments. The values (mean ± SD, *N* = 3) represent the ATG5/Actin ratio and are normalized for the results obtained after treatment with vehicle alone (leftmost lane) whose ATG5/Actin value is taken as 1.0. R.I. = Relative Intensity.

As no data on the use of ethanolamine in myeloid cells are available, we performed preliminary experiments to define the maximal tolerated concentrations of ethanolamine. In *NB4* cells, ethanolamine concentrations above 50 µM are significantly cytotoxic. Thus, we treated the cells with two non-toxic concentrations of ethanolamine (20 μM and 50 μM) for 24 h before exposure to vehicle or ATRA for a further 48 h. In these conditions, ethanolamine does not alter the viability of *NB4* cells observed in either the absence or presence of ATRA (Fig. 3A). In contrast, the two concentrations of ethanolamine cause a mild anti-proliferative action in cells exposed to vehicle. This last effect and the well-known growth inhibition triggered by ATRA in *NB4* cells are non-additive, suggesting that the anti-proliferative action of the two compounds is activated by a similar mechanism (Fig. 3A). Neither ATRA nor ethanolamine alone or in combination alter the mitochondrial mass, as indicated by Mito-Tracker green-fluorescence (Fig. 3B) and citrate synthase activity (Supplementary Fig. S3). More importantly, the highest concentration of ethanolamine blocks the decrease in mitochondrial activity caused by ATRA, as indicated by the Mito-Tracker red/green-fluorescence values (Fig. 3B). Consistent with this, the two concentrations of ethanolamine block the ATRA-dependent decrease of mitochondrial Complex-I, Complex-III and Complex IV activities (Fig. 3C).

To define whether the protective effects exerted by ethanolamine on mitochondrial activity are accompanied by perturbations of the retinoid-dependent differentiation, we determined the myeloid markers, *NBT-reductase* activity [13], CD11b and CD11c (Fig. 3D). At 20 μM and 50 μM, ethanolamine reduces the increase in *NBT-reductase* activity and CD11b expression observed with ATRA. *NB4* cell exposure to the highest concentration of ethanolamine is also associated with an attenuation in the ATRA-dependent increase of CD11c. The suppressive action of ethanolamine on CD11b and CD11c is specific, as the compound does not affect CD33, a myeloid marker which is not modulated by ATRA in APL cells [13, 26]. Further support to the idea that ethanolamine inhibits the granulocytic differentiation process ignited by ATRA in *NB4* cells comes from the measurement of two other markers, PU.1 and IRF1, which are transcription-factors involved in the control of myeloid maturation [27, 28]. Indeed, ethanolamine suppresses ATRA-dependent PU.1/IRF1 induction (Fig. 3E).

As autophagy plays an important role in the ATRA-dependent differentiation of *NB4* and APL cells, *via* degradation of PML-RARα [29–31], we determined the effects exerted by ethanolamine on this process by measuring the autophagy-related ATG5 [32] and LC3I/II [33] proteins. Ethanolamine causes a dose-dependent inhibition of ATRA-dependent ATG5 induction (Fig. 3F). The suppressive action of ethanolamine is more evident in the case of LC3I/II which are detectable only in *NB4* cells exposed to ATRA alone. Autophagy inhibition does not involve PML-RARα and/or RARα, as ethanolamine has no effect on the basal levels or the ATRA-dependent degradation of either protein (Supplementary Fig. S4).

Our data support the idea that *CLs* down-regulation is at the basis of the ATRA-dependent decrease in mitochondrial activity and contributes to the differentiation/autophagy processes stimulated by the retinoid.

### ATRA-dependent action on genes controlling CLs biosynthesis and autophagy in NB4 cells

To define the mechanisms underlying the action of ATRA on *CLs*, mitochondrial homeostasis and autophagy, we performed *RNA-seq* (RNA-sequencing) studies in *NB4* cells. Exposure of these cells to ATRA for 48 h results in a significant (FDR < 0.05) up- and down-regulation of 4066 and 3605 genes (Supplementary Table. S2).

**Fig. 4:**
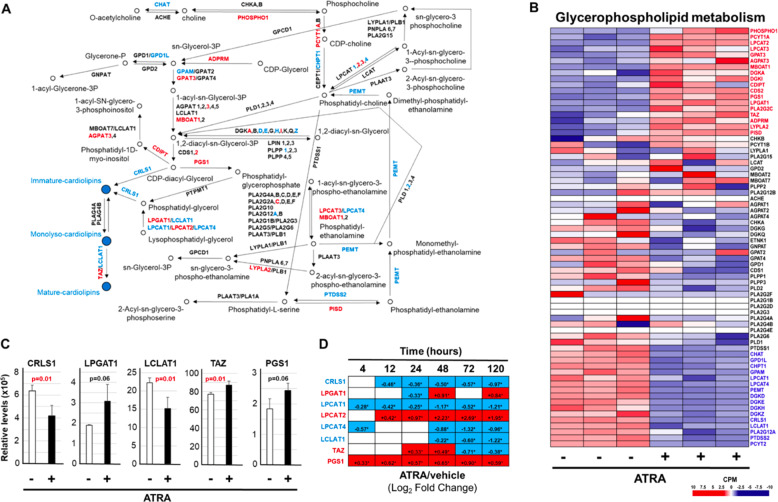
ATRA modulates the expression of genes controlling the biosynthesis and degradation of glycerophospholipids in NB4 cells. The effects exerted by ATRA on the genes involved in the metabolism of gycerophospholipids were evaluated using the *RNA-seq* data generated from *NB4* cells exposed to vehicle (DMSO) or ATRA (1 μM) for 48 h. **A** The panel shows a schematic diagram of the human “Glycerophospholipid Metabolism” pathway. The figure is based on the information available in the corresponding KEGG pathway (hsa00564). When the mRNAs encoding the proteins catalyzing the indicated metabolic reactions, are up- and down-regulated by ATRA [*RNA-seq* data shown in (**B**)] they are marked in red and blue, respectively. **B** The heat-map shows the absolute expression levels of the indicated genes in the three biological replicates of the *NB4* cell cultures exposed to vehicle or ATRA. The heat-map includes the genes of the “glycerophospholipid metabolism” pathway identified with the *RNA-seq* data. The names of the genes whose expression is significantly up-regulated by ATRA are written in red (FDR < 0.05). The names of the genes whose expression is significantly down-regulated by ATRA are written in blue (FDR < 0.05). The names of the genes whose expression is unaffected by ATRA are written in black. **C** The panel illustrates the validation of the *RNA-seq* data shown in (**B**) obtained by PCR analysis of the indicated genes, using specific Taqman assays. Each column represents the Mean + SD of the expression values determined in three independent cell cultures treated with vehicle or ATRA (1 μM) for 48 h. The *p* value of the ATRA vs. vehicle comparison (unpaired Student’s *t* test) is shown and it is marked in red when it is significant. **D** The table shows the time course of the perturbations induced by ATRA on the expression of the indicated genes. The results are expressed using the Log_2_ of the ATRA-dependent Fold-Change value determined on the basis of the data published in the literature (4,12, 24, 72, and 120 h) and the *RNA-seq* data (48 h) shown in (**B**). When the indicated genes are significantly up-regulated (FDR > 0.05), the positive fold-change values are contained in a red box and are marked with an asterisk. When the indicated genes are significantly down-regulated (FDR > 0.05), the negative fold-change values are contained in a blue box.

Initially, we focused our attention on genes involved in the synthesis/degradation/remodelling of *CLs* [21], a process which is a part of the KEGG “*Glycerophospholipid metabolism*” pathway (Fig. 4A). Thus, we evaluated the effects exerted by ATRA on the expression of the mRNAs coding for the 70 enzymes belonging to this pathway using our *RNA-seq* results. ATRA up- and down-regulates 18 and 16 mRNAs, respectively (Fig. 4B). As for the transcripts coding for proteins involved in *CLs* metabolism, ATRA up-regulates PGS1 (phosphatidylglycerophosphate-synthase-1), which catalyses the biotransformation of CDP-diacyl-glycerol into phosphatidyl-glycerophosphate (Fig. 4A). By converse, ATRA down-regulates various transcripts coding for enzymes laying downstream along the *CLs* synthetic pathway. The retinoid reduces CRLS1 (cardiolipin-synthase-1) mRNA, whose protein-product transforms CDP-diacyl-glycerol and phosphatidyl-glycerol into immature *CLs*, as well as LPCAT1/LPCAT4 (lysophosphatidylcholine-acyltransferases-1/-4) and LCLAT1 (lysocardiolipin-acyl-transferase-1) which metabolize lysophosphatidyl-glycerol into phosphatidyl-glycerol (Fig. 4A). Noticeably, LCLAT1 is also involved in *CLs* remodelling, transforming monolyso-*CLs* into the mature counterparts (Fig. 4A). The CRLS1/LCLAT1 down-regulation and TAZ/PGS1/LPGAT1 up-regulation are validated by real-time PCR experiments (Fig. 4C).

Subsequently, we determined the time-frame of the perturbations caused by ATRA on CRLS1/LPGAT1/LPCAT1/LPCAT2/LPCAT4/LCLAT1 and PGS1. Thus, we compared our *RNA-seq* data with the publicly available transcriptomic results obtained in *NB4* cells exposed to ATRA for 4, 12, 24, 72, and 120 h [34, 35] (Fig. 4D). LPCAT1/CRLS1 down-regulation are early and long-lasting events, as they are already observed at 4/12 h and are maintained until 120 h. In contrast, ATRA-dependent LCLAT1 down-regulation is a late event observed only at 72 and 120 h. The ATRA-induced decrease in the levels of LPCAT4 mRNA is a biphasic process characterized by a short-lived up-regulation at 4 h and a late/long-lasting down-regulation which starts to be evident at 48 h. As for the up-regulated mRNAs, ATRA-dependent PGS1 and LPCAT2 induction are early and long-lived events, which are already evident at 4/12 h. The action of ATRA on TAZ is biphasic, as the up-regulation observed at 24/48 h is followed by a down-regulation. Overall, our results support the idea that the early and ATRA-dependent decrease in CRLS1/LPCAT1 and increase in PGS1 expression may be causally related to the reduction in the amounts of *CLs* observed in *NB4* cells. The relevance of the perturbations afforded by ATRA on the expression of CRLS1, LPCAT1 and PGS1 mRNAs is sustained by the fact that this translates into the expected down-regulation (CRLS1 and LPCAT1) and up-regulation (PGS1) of the corresponding proteins in retinoid-sensitive *NB4* cells, but not in retinoid-resistant *NB4.306* cells (see Fig. 8C).

As the ATRA-induced effects on *CLs* are accompanied by a decrease in the activity of mitochondria, we evaluated our *RNA-seq* data for the expression of the 1276 mRNAs encoding mitochondrial proteins (Gene Ontology database, *GO*_Cellular Components). *NB4* cells exposure to ATRA for 48 h causes a significant down-regulation and up-regulation of 381 and 296 transcripts, respectively (Supplementary Table. S3). Incidentally, 8 of the ATRA-induced RNAs coding for mitochondrial proteins are the products of genes originating from mitochondrial DNA and their expression is only slightly increased by the retinoid (Fold Change < 1.7). These last observations are consistent with the fact that ATRA does not exert major effects on the mitochondrial mass. In addition, the results obtained on the genes coding for mitochondrial proteins indicate that the ATRA-dependent reduction in the activity of this organelle is not explained by transcriptomic effects and may be due to the decrease in the levels of *CLs*.

**Fig. 5:**
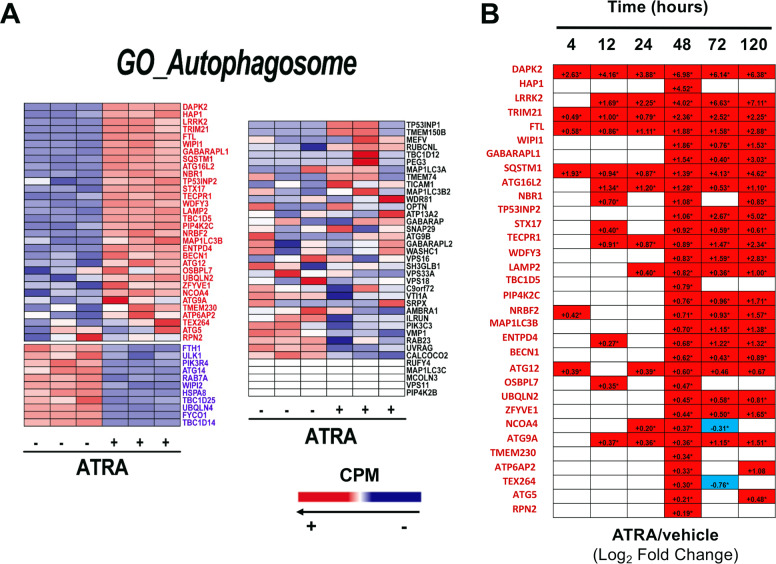
ATRA effects on the expression of the genes belonging to the GO_Autophagosome network in NB4 cells. **A** The effects exerted by ATRA on the genes belonging the *GO_Autophagosome* network were evaluated using the *RNA-seq* data generated from *NB4* cells exposed to vehicle (DMSO) or ATRA (1 μM) for 48 h. The heat-maps show the absolute expression levels of the indicated genes in the three biological replicates of the *NB4* cell cultures exposed to vehicle or ATRA. The data are expressed in counts per million (CPM). The names of the genes whose expression is significantly up-regulated by ATRA are written in red (FDR < 0.05). The names of the genes whose expression is significantly down-regulated by ATRA are written in blue (FDR < 0.05). The names of the genes whose expression is unaffected by ATRA are written in black. **B** The table shows the time course of the perturbations induced by ATRA on the expression of the 32 genes belonging to the *GO_Autophagosome* network which are significantly up-regulated by ATRA, as shown in (**A**). The results are expressed using the Log_2_ of the ATRA-dependent fold-change value determined on the basis of the data published in the literature (4,12, 24, 72, and 120 h) and the *RNA-seq* data (48 h) shown in (**A**). When the indicated genes are up-regulated the positive fold-change values are contained in a red box. When the indicated genes are down-regulated the negative fold-change values are contained in a blue box.

The data obtained with ethanolamine (Fig. 3F) indicate that the compound suppresses the ATRA-stimulated process of autophagy, which is involved in *NB4* differentiation [31]. Hence, we performed pathway-enrichment analysis of the *RNA-seq* data (Supplementary Table. S2), identifying five *GO*_networks involved in autophagy which are significantly up-regulated by ATRA (*GO_Autophagosome*;*GO_Autophagosome_Membrane*;*GO_Macroautophagy*;*GO_Process_Utilizing_Autophagic_Mechanism*;*GO_autophagosome_organization*). Focussing on “*GO_Autophagosome*”, the *RNA-seq* data indicate that *NB4* cells express measurable amounts of 75 of the 98 genes belonging to this gene-network (Fig. 5A). At 48 hours, ATRA causes a significant up- and down-regulation of 32 and 11 genes, respectively, which is consistent with an overall activation of the autophagic process. As for the 32 up-regulated genes, we evaluated the time-frame of the ATRA-dependent effects, using the publicly available transcriptomic data. Up-regulation of ~30% of these autophagic genes (14/32) occurs early (4, 12, or 24 h), and it is long-lasting, since it is maintained at later time-points (Fig. 5B).

### Effects of autophagy inhibition on the ATRA-dependent decrease of CLs and mitochondrial activity

**Fig. 6:**
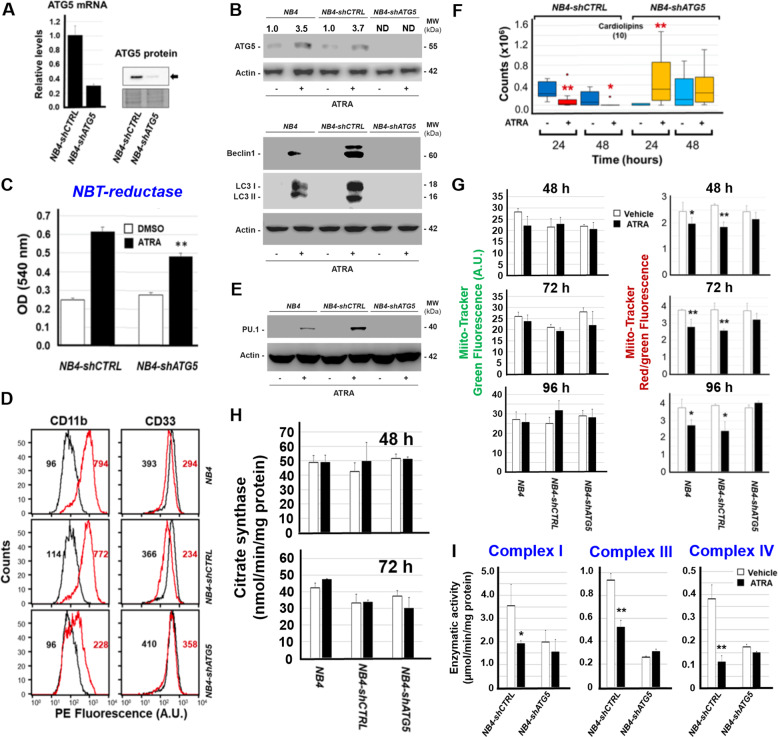
Silencing of the ATG5 gene in NB4 cells. Parental *NB4* cells were infected with a mixture of two lentiviral constructs allowing the expression of shRNAs targeting ATG5 (*shATG5*) or a control non-targeting construct (*shCTRL*). The corresponding cellular populations (*NB4-shATG5* and *NB4-shCTRL*) were isolated. **A** The left panel shows the levels of the ATG5 mRNA in three independent cultures of *NB4-shCTRL* and *NB4-shATG5* cells, as determined by PCR using a specific Taqman assay. The results are expressed as the Mean + S.D. (*N* = 3) of the values determined. The right panel shows the levels of the ATG protein in the two cell populations which were determined by Western blot analysis (upper) and the amounts of protein loaded in each lane as determined by Ponceau red staining of the blot. The Western blot analysis was performed on pooled cellular extracts obtained from the three independent cultures described above, using antibodies targeting the autophagic marker ATG5. **B** The panel shows the Western blot analysis of the indicated proteins which were performed with total cellular extracts obtained from 3 independent cultures of parental *NB4*, *NB4-shCTRL*, and *NB4-shATG5* cells exposed to vehicle (DMSO) or ATRA (1 μM) for 48 h. The Western blot analyses were performed on pooled cellular extracts obtained from the three independent cultures mentioned above. The same amount of protein was loaded in each lane, as indicated by the amount of β_2_-actin (Actin) determined. The Western blots shown are representative of three independent experiments producing similar results. **C** *NBT (Nitro-Blue-Tetrazolium)-reductase* activity was determined on cell extracts obtained from three independent cultures of the indicated cell populations exposed to vehicle (DMSO) or ATRA (1 μM) for 48 h. Each value is the mean + S.D. of three samples. **Significantly lower than the corresponding *NBT*-reductase value determined in *NB4-shCTRL* cells exposed to ATRA (*p* < 0.01, unpaired Student’s *t* test). **D** FACS analyses of the CD11b and CD33 surface markers performed in parental *NB4*, *NB4-shCTRL*, and *NB4-shATG5* cells exposed to vehicle (black graphs) or ATRA (red graphs) as in (**C**). The indicated values represent the AUC (area-under-the-curve) of the corresponding graphs. The FACS analyses shown are representative of two independent experiments producing similar results. **E** Western blot analysis performed on pooled cellular extracts obtained from three independent cultures of parental *NB4*, *NB4-shCTRL*, and *NB4-shATG5* cells exposed to vehicle (DMSO) or ATRA (1 μM) for 48 h, using antibodies targeting the differentiation markers, PU.1. The same amounts of proteins were loaded in each lane, as demonstrated by the Western blot signals obtained with the anti-β_2_actin (Actin) antibodies. The Western blots shown are representative of two independent experiments producing similar results. **F** Biological triplicates of the indicated *NB4-shCTRL* and *NB4-shATG5* cells were treated with vehicle (DMSO) or ATRA (1 μM) for 24 and 48 h. The box plots show the median ± SD levels of *CLs*. The number of different molecules identified by mass-spectrometry is indicated in parenthesis. **Significantly different relative to the corresponding vehicle treated control (*p* < 0.01, unpaired Student’s *t* test). *Significantly different relative to the corresponding vehicle treated control (*p* < 0.05, unpaired Student’s *t* test). **G** Three independent cultures of parental *NB4*, *NB4-shCTRL*, and *NB4-shATG5* cells were treated with ATRA (1 μM) for 48, 72, and 96 h. At the end of the treatment cells were co-stained with MitoTracker-green and MitoTracker-red dyes before subjecting them to FACS analysis. The left diagrams show the levels of Mito-Tracker green fluorescence, while the right diagrams show the values of MitoTracker-red/green fluorescence (red fluorescence following normalization for the green fluorescence value). Each value is the mean + S.D. of three samples. **Significantly different relative to the corresponding vehicle treated control (*p* < 0.01, unpaired Student’s *t* test). *Significantly different relative to the corresponding vehicle treated control (*p* < 0.05, unpaired Student’s *t* test). The diagrams shown are representative of two independent experiments producing similar results. **H** Effects of ATG5 silencing on citrate synthase activity in cells exposed to ATRA: three independent cultures of parental *NB4*, *NB4-shCTRL*, and *NB4-shATG5* cells were treated with ATRA (1 μM) for 48 and 72 h. At the end of the treatment, citrate synthase activity was measured in cell homogenates. Each value is the Mean + S.D. of the three independent cultures. **I** Effects of ATG5 silencing on mitochondrial Complex I, Complex III, and Complex IV enzymatic activity in cells exposed to ATRA: mitochondria were isolated from pools of three independent cultures of *NB4-shCTRL* and *NB4-shATG5* cells exposed to vehicle or ATRA (1 µM) for 48 h. Mitochondrial Complex I, Complex III, and Complex IV enzymatic activities were measured on five replicates and the results are expressed as the Mean + SD. The data are representative of two independent experiments providing similar results. **Significantly lower than the corresponding vehicle treated control (*p* < 0.01, unpaired Student’s *t* test).

To define the role of autophagy in the processes activated by ATRA, we generated a *NB4*-derived cell-population (*NB4-shATG5*) silenced for ATG5, an autophagy mediator [36], and a control cell-population bearing a non-targeting shRNA construct (*NB4-shCTRL*). Relative to *NB4-shCTRL* cells, *NB4-shATG5* cells show a reduction in the basal levels of ATG5 mRNA and protein (Fig. 6A). In *NB4-shCTRL* and parental *NB4* cells, we also observe a suppression of the ATRA-dependent induction of the ATG5 protein (Fig. 6B) and an inhibition of the autophagic response triggered by the retinoid (see the two autophagic markers, Beclin1 and LC3I/II). Inhibition of the autophagic response is accompanied by a reduction of the granulocytic maturation induced by ATRA [31]. Indeed, ATRA stimulates *NBT-reductase* activity (Fig. 6C), CD11b (Fig. 6D), and PU.1 (Fig. 6E) expression in parental *NB4* and/or *NB4-shCTRL* cells, while ATRA-dependent stimulation of these myeloid markers is diminished/abolished in *NB4-shATG5* cells.

Subsequently, we evaluated the lipidomic profiles of *NB4-shATG5* and *NB4-shCTRL* cells exposed to vehicle or ATRA for 24/48 h, identifying 16 distinct sub-classes of lipids (Supplementary Table. S4 and Supplementary Fig. S5A). In *NB4-shATG5* and *NB4-shCTRL* cells, 9 of these lipid sub-classes are the same as in *NB4* and *NB4.306* cells. Once again, the effects exerted by ATRA on the lipid composition of *NB4-shATG5* and *NB4-shCTRL* cells are limited, as indicated by the PCA results (Supplementary Fig. S5B). In particular, ATRA does not alter the levels of five lipid sub-classes in either *NB4-shCTRL* or *NB4-shATG5* cells (Supplementary Fig. S6). Consistent with the results obtained in *NB4* cells (Fig. 1), the exposure of *NB4-shCTRL* cells to ATRA for 48 h tends to decrease the levels of *diacylglycerophosphocholines* and *monoacylglycerophosphocholines*. Similar effects are not observed in *NB4-shATG5* cells, where the amounts of *monoacylglycerophosphocholines* are actually increased by the retinoid at 24 h. The action of ATRA on other lipid sub-classes is variable and it is evident only in *NB4-shCTRL* cells. As observed in *NB4* cells (Fig. 1), ATRA causes a significant down-regulation of *CLs* in *NB4-shCTRL* cells at 24/48 h, while similar effects are not evident in *NB4-shATG5* cells (Fig. 6F).

To establish whether the suppression of *CLs* down-regulation has consequences on the mitochondrial mass/function, we exposed *NB4*, *NB4-shCTRL* and *NB4-shATG5* cells to ATRA for 48, 72, and 96 h before Mito-Tracker staining (Fig. 6G). As indicated by Mito-Tracker green-fluorescence, ATRA does not alter the mass of mitochondria in *NB4*, *NB4-shCTRL* and *NB4-shATG5* cells at any time point. This is supported by the measurement of citrate synthase at 48 and 72 h (Fig. 6H). In contrast, exposure of *NB4* and *NB4-shCTRL* cells to ATRA for 48, 72, and 96 h decreases mitochondrial function (Fig. 6G, Mito-Tracker red/green-fluorescence). Mito-Tracker red/green-fluorescence is not modified by the retinoid in *NB4-shATG5* cells. Consistent with this, exposure of *NB4-shATG5* cells to ATRA for 48 h is devoid of any effect on mitochondrial Complex-I, Complex-III, and Complex-IV activities (Fig. 6I), which are significantly reduced in *NB4-shCTRL* and parental *NB4* cells. Thus, autophagy inhibition suppresses not only the ATRA-dependent down-regulation of *CLs*, but also the corresponding decrease in mitochondrial function.

### Autophagy-inhibition and ATRA-dependent gene-expression

**Fig. 7:**
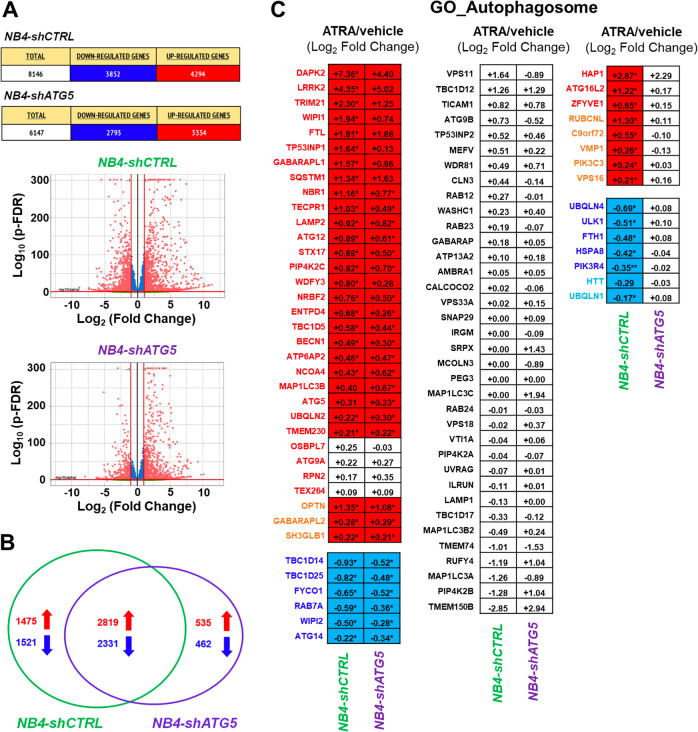
Gene-expression perturbations afforded by ATRA in NB4-shCTRL and NB4-shATG5 cells. The effects exerted by ATRA on the transcriptomic profiles of *NB4-shCTRL* and *NB4-shATG5* cells were evaluated using the *RNA-seq* data generated from cells exposed to vehicle (DMSO) or ATRA (1 μM) for 48 h. **A** The upper section of the panel illustrates the total number of genes up- or down-regulated by ATRA in *NB4-shCTRL* and *NB4-shATG5* cells. The lower Volcano plots indicate the effects exerted by ATRA on the expression levels of all the identified genes in the three biological replicates of *NB4-shCTRL* and *NB4-shATG5* cells exposed to vehicle or ATRA. The genes whose expression is significantly up-regulated or down-regulated by ATRA are marked in red (FDR < 0.05). The genes whose expression is not significantly modified by ATRA are marked in blue (FDR > 0.05). **B** The panel shows a VENN diagram illustrating the number of genes selectively or commonly up-regulated (red) and down-regulated (blue) by ATRA in *NB4-shCTRL* and *NB4-shATG5* cells. **C** The diagrams show the perturbations induced by ATRA on the expression of the 89 genes belonging to the *GO_Autophagosome* network. The results presented in each cell are expressed using the Log_2_ of the ATRA-dependent Fold-Change values which we determined. The names of the genes whose expression is significantly increased by ATRA in *NB4-shCTRL* and parental *NB4* cells (see Fig. 5) are written in red. The names of the genes whose expression is significantly increased by ATRA in *NB4-shCTRL*, but not in parental *NB4* cells (see Fig. 5), are written in orange. The names of the genes whose expression is significantly decreased by ATRA in *NB4-shCTRL* (see Fig. 5) are written in dark blue. The names of the genes whose expression is significantly decreased by ATRA in *NB4-shCTRL*, but not in parental *NB4* cells (see Fig. 5), are written in light blue. If a significant up- or down-regulation of the indicated genes is not observed in *NB4-shCTRL* or in *NB4-shATG5* and parental *NB4* cells (see Fig. 5) cells, the names of the genes are written in black and they are contained in a white cell. The genes showing up- or down-regulation or no regulation by ATRA in both *NB4-shCTRL* and *NB4-shATG5* cells are shown on the left. The genes whose expression is not altered by ATRA in either *NB4-shCTRL* and *NB4-shATG5* cells are shown in the middle. The genes whose expression is significantly up- or down-regulated in *NB4-shCTRL* but not in *NB4-shATG5* cells are shown on the right.

To establish whether autophagy plays any role in the ATRA-dependent expression of genes controlling glycerophospholipid metabolism, we performed *RNA-seq* studies in *NB4-shCTRL* and *NB4-shATG5* cells exposed to the retinoid for 48 h (Supplementary Table. S5). In *NB4-shCTRL* and *NB4-shATG5* cells, ATRA modifies the expression of ~8000 and 6000 genes, respectively (Fig. 7A). In *NB4-shCTRL* cells, ATRA selectively up- or down-regulates ~3000 genes, while the number of selectively modulated genes falls to ~1000 in *NB4-shATG5* cells (Fig. 7B). We evaluated the perturbations afforded by inhibition of the autophagic process on the ATRA-dependent expression profiles of the genes belonging to the *GO_Autophagosome* pathway. In *NB4-shCTRL* cells, ATRA up-regulates 28 of the 32 mRNAs (Fig. 7C, upper left and right diagrams) which show a similar up-regulation in parental *NB4* cells (Fig. 5A). Eight of the mRNAs left unaltered in *NB4* cells are up-regulated by ATRA in *NB4-shCTRL* cells (Fig. 7C, upper-left/upper-right diagrams). In *NB4-shCTRL* cells, ATRA down-regulates all the 11 mRNAs (Fig. 7C, lower-left/lower-right diagrams) which are also down-regulated in parental *NB4* cells (Fig. 5A). Only two of the mRNAs whose expression is left unaltered in *NB4* cells, are down-regulated by ATRA in *NB4-shCTRL* cells. As for the above 28 up-regulated mRNAs, only ATRA-dependent induction of ATG16L2, ZFYVE1 and HAP1 is suppressed in *NB4-shATG5* cells (Fig. 7C, upper-right diagram). As for the 11 down-regulated mRNAs, ATG5-silencing restores the levels ULK1, FTH1, PIK3R4, UBQLN4, and HSPA8 (Fig. 7C, lower-right diagram).

**Fig. 8:**
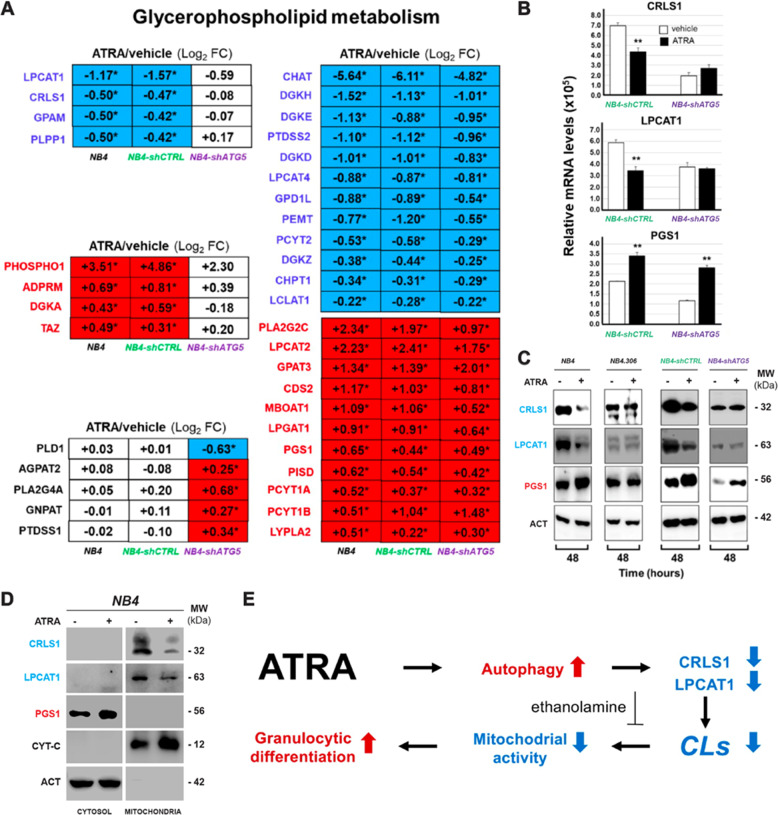
ATRA effects on the expression of the genes belonging to the glycerophospholipid metabolism pathway in parental NB4, NB4-shCTRL, and NB4-shATG5 cells. **A** The effects exerted by ATRA on the genes belonging to the glycerophospholipid pathway in parental *NB4*, *NB4-shCTRL*, and *NB4-shATG5* cells were evaluated using the *RNA-seq* data generated from the three cell lines exposed to vehicle (DMSO) or ATRA (1 μM) for 48 h. The diagrams show the perturbations induced by ATRA on the expression of the indicated genes. The results presented in each box are expressed using the Log_2_ of the ATRA-dependent fold-change values which we determined. The names of the genes whose expression is significantly increased by ATRA in the indicated cell-lines are written in red. The names of the genes whose expression is significantly decreased by ATRA in the indicated cell-lines are written in blue. When the indicated genes are significantly up-regulated (FDR > 0.05), the positive fold-change values are contained in a red box and are marked with an asterisk. When the indicated genes are significantly down-regulated (FDR > 0.05), the negative fold-change values are contained in a light blue box. **B** The column diagrams indicate the expression levels of the *CRLS1*, *LPCAT1*, and *PGS1* mRNAs which were determined in three independent cultures of *NB4-shCTRL* and *NB4-shATG5* cells, by PCR using specific Taqman Assays. The results are expressed as the Mean + S.D. (*N* = 3) of the values determined. **Significantly different relative to the corresponding vehicle-treated control (*p* < 0.01, unpaired Student’s *t* test). The diagrams shown are representative of two independent experiments producing similar results. **C** Western blot analyses were performed on pooled cellular extracts obtained from three independent cultures of parental *NB4*, *NB4.306*, *NB4-shCTRL*, and *NB4-shATG5* cells exposed to vehicle (DMSO) or ATRA (1 μM) for 48 h. The Western blots were performed with the use of specific antibodies targeting the indicated proteins. The same amounts of proteins were loaded in each lane, as demonstrated by the Western blot signals obtained with the anti-β_2_actin (ACT) antibodies. The Western blots shown are representative of two independent experiments producing similar results. **D** The Western blots summarize the effects of ATRA on the expression of *CRLS1* and *LPCAT1* proteins in the cytosolic and mitochondrial fractions of *NB4* cells exposed to vehicle or ATRA. Three independent cultures of *NB4* cells were treated with vehicle (DMSO) or ATRA (1 µM) for 48 h. At the end of the treatment the mitochondrial and cytosolic fractions of the cell homogenates were isolated. The mitochondrial and cytosolic fractions were pooled and subjected to Western blot analysis with antibodies targeting *CRLS1*, *LPCAT1*, *PGS1*, cytochrome-c (*CYT-C*) and actin proteins, as indicated. The same amount of cytosolic and mitochondrial proteins was loaded in each couple of lanes (vehicle and ATRA). The Western blots shown are representative of two independent experiments producing similar results. **E** The scheme summarizes the effects exerted by ATRA in APL blasts, which lead to terminal granulocytic maturation of the *NB4* cells.

We further assessed whether autophagy controls ATRA-dependent genes involved in “*glycerophospholipid-metabolism*” with particular reference to *CLs*. The majority of the “*glycerophospholipid-metabolism*” genes which are down-regulated (12/16) or up-regulated (11/15) in *NB4* and *NB4-shCTRL* cells are similarly modulated by ATRA in *NB4-shATG5* cells (Fig. 8A, right). In contrast, ATRA-dependent down-regulation of CRLS1/LCLAT1/GPAM/PLPP1 mRNAs and ATRA-dependent up-regulation of ADPRM/DGKA/PHOSPHO1/TAZ mRNAs are suppressed in *NB4-shATG5* cells (Fig. 8A, left). This indicates that altered expression of the eight genes is mediated by ATRA-stimulated autophagy.

As autophagy-dependent CRLS1/LPCAT1 down-regulation is likely to be of significance for *CLs* biosynthesis/maturation, we determined the levels of the corresponding mRNAs and proteins in *NB4-shCTRL* and *NB4-shATG5* cells exposed to ATRA for 48 h. Real-time PCR-analysis of the CRLS1/LPCAT1 mRNAs confirms the results obtained by *RNA-seq* (Fig. 8B). In addition, we validate the ATRA-dependent PGS1up-regulation in *NB4-shCTRL* and *NB4-shATG5* cells. In *NB4-shCTRL* cells, down-regulation of the CRLS1/LPCAT1 transcripts is accompanied by a reduction in the levels of the corresponding proteins, which is abolished in *NB4-shATG5* cells (Fig. 8C). In contrast, the PGS1 protein is increased in both *NB4-shCTRL* and *NB4-shATG5* cells. In parental *NB4* and *NB4-shCTRL* cells, the ATRA-dependent reduction of CRLS1/LPCAT1 proteins is already evident at 24 h (data not shown).

To evaluate whether an ATRA-dependent down-regulation of CRLS1/LPCAT1 proteins is observed in mitochondria where the two enzymes exert their action, we performed Western blot experiments in mitochondrial and cytosolic fractions of *NB4* cells exposed to vehicle or ATRA (1 µM) for 48 h (Fig. 8D). We used the cytosolic PGS1 and the mitochondrial cytochrome-c proteins as controls. ATRA causes a significant down-regulation of mitochondrial CRLS1 and LPCAT1 proteins relative to what is observed in the vehicle treated counterparts, while the two enzymes are never detectable in the cytosol of *NB4* cells. These observations strengthen the concept that CRLS1 and LPCAT1 down-regulation is involved in the ATRA-dependent reduction of CL levels (Fig. 8E).

## Discussion

We used the *NB4* model [37] to define the effects exerted by ATRA on lipid homeostasis, performing non-oriented lipidomic studies [18, 38]. In fact, this global lipid profiling approach has been successfully applied in early stage pre-clinical work and clinical investigations [39–41]. Our data indicate that ATRA causes an early and long-lasting decrease in the levels of mitochondrial *CLs*. In *NB4* cells, ATRA-dependent down-regulation of *CLs* is evident before the appearance of any sign of granulocytic differentiation. A similar decrease in the amounts of *CLs* is not observed in retinoid-resistant *NB4.306* cells [13, 16], suggesting that *CLs* down-regulation is causally involved rather than the consequence of ATRA induced granulocytic maturation.

*CLs* play an important role in mitochondrial homeostasis, as these glycerophospholipids facilitate the assembly of the respiratory chain super-complexes [42, 43]. Consistent with this, ATRA-dependent down-regulation of *CLs* reduces mitochondrial activity in *NB4* cells. Noticeably, ATRA-treated *NB4* cells show an almost concomitant decrease in *CLs* levels and mitochondrial activity. The observation suggests that the mitochondrial dysfunction associated with *CLs* diminution precedes and contributes to the process of *NB4* differentiation ignited by ATRA. The idea is supported by the fact that ethanolamine suppresses the ATRA-dependent inhibition of mitochondrial activity and granulocytic maturation observed in *NB4* cells. In line with the idea that autophagy activation is involved in myeloid differentiation [31, 44], ethanolamine suppresses also the retinoid-dependent induction of the autophagy markers, ATG5, beclin, and LC3I/II.

In *NB4* blasts, it is difficult to establish the causal relationship linking *CLs* downregulation, mitochondrial dysfunction and stimulation of the autophagic response, as the three processes are activated by ATRA almost contemporaneously. However, the data obtained in *NB4-shATG5* cells, which are characterized by suppression of ATRA-dependent autophagy/differentiation, clarify the issue. Indeed, exposure of *NB4-shATG5* cells to ATRA does not cause the *CLs* down-regulation and the mitochondrial dysfunction observed in parental *NB4* and *NB4-shCTRL* cells. This is consistent with the idea that ATRA-dependent activation of the autophagic process causes *CLs* down-regulation, which, in turn, is responsible for the reduction of mitochondrial functionality.

The transcriptomic data obtained in *NB4*, *NB4-shCTRL*, and *NB4-shATG5* cells provide insights into the molecular mechanisms underlying the downregulation of *CLs* resulting from ATRA-stimulated autophagy. In *NB4* and *NB4-shCTRL* cells, ATRA-induced autophagy results in the down-regulation of CRLS1 and LPCAT1, which catalyze the last steps of *CLs* biosynthesis. Consistent with an autophagy mediated and indirect effect of ATRA on *CLs* homeostasis, a similar down-regulation of CRLS1 and LPCAT1 is not observed in *NB4-shATG5* cells.

## Materials And Methods

### Cell cultures

*NB4* and *NB4.306* cell-lines were maintained in RPMI-1640 medium containing 10% fetal-calf serum (GIBCO). The parental *NB4*, *NB4.306*, and *NB4*-derived cell-lines were mycoplasma-free. *NB4* and *NB4.306* cells were authenticated by karyotype, morphology, and *RNA-seq* analysis. Cell growth and cell viability were evaluated with a Cell Viable Analyzer (Vi-cell_XR_; Beckman Coulter Life Sciences, Milano, Italy).

### Generation of the NB4-shCTRL and NB4-shATG5 cells

The pLKO.1-puro lentiviral vectors expressing ATG5-targeting shRNAs (shATG5_mix: NM_004849.1–420s1c1/TRCN0000150940; NM_004849.1–915s1c1/TRCN0000151474) and a control shRNA (SHC002) were from Sigma-Aldrich. Lentivirus production and transduction were performed as described [45]. Briefly, the third-generation, packaging plasmids *pMD.G* (VSV-G), *pMDLg/p.RRE* (gag and pol), and *pRSV-Rev* (Rev gene), as well as the lentiviral transfer vector (with the shRNA) were delivered to *293* *T* cells using the calcium-precipitation method. Sixteen hours later, medium was changed. After a further 48 h, viral supernatants were harvested and filtered. *NB4* cells were transduced twice with 500 µl of filtered, virus-containing supernatant. As the lentiviral vectors express a puromycin resistance gene, 2 days post-infection, we selected the infected cell populations with 1.5 μg/ml puromycin for 4 days before lowering the concentration of the compound to 0.5 μg/ml.

### RNA-seq studies

Parental *NB4*, *NB4-shCTRL*, and *NB4-shATG5* cells were grown in RPMI-1640 medium containing 10% fetal-calf serum (Fetal-calf Serum, Gibco) for 24 h. Cells were treated with vehicle (DMSO) or ATRA (1 μM) for another 48 h. The *RNA-seq* data were generated and analyzed as follows. Total RNA was extracted with the miRNeasy Mini Kit (QIAGEN, Hilden, Germany). cDNA libraries were prepared with the Illumina TruSeq RNAlibrary preparation kit (Illumina, San Diego, CA, USA). RNA-sequencing was performed on the Illumina NextSeq500 with paired-end, 121 base pair long, reads. The overall quality of sequencing reads was determined using the FastQC protocol [46] (http://www.bioinformatics.babraham.ac.uk/projects/fastqc/). Sequence alignments to the reference human genome (GRCh38) were performed using STAR (v.2.5.2a). Gene-expression was quantified at the single gene level using the comprehensive annotations made available by Gencode [47]. In particular, we used the v27 release of the Gene Transfer File.

Raw-counts were further processed in the R Statistical environment and downstream differential expression analysis was performed using the DESeq2 pipeline. Genes characterized by low mean normalized counts were filtered out automatically by the Independent Filtering feature embedded in DESeq2 (alpha = 0.05). All the statistical analyses were corrected for multiple comparisons, using the Benjamini–Hochberg correction method (FDR). DESeq2-computed Wald-statistics values were used as input for gene-set enrichment testing performed with the pre-ranked version of Camera (inter-gene correlation equal to 0.1, non-parametric test procedure). Statistical enrichments were determined for gene-sets obtained from the Gene Ontology (GO) collection (c5), which are curated by the Molecular Signature DataBase (MSigDB) [48]. All the *RNA-seq* data were deposited in the EMBL-EBI Arrayexpress database (Accession No: E-MTAB-10267).

### Lipidomic studies

Untargeted lipidomics studies were performed with Lipostar, a high-throughput software used for targeted and untargeted liquid-chromatography/mass-spectrometry (LC-MS) lipidomics [18]. Briefly, lipids were extracted from the cell samples by using an appropriate volume (1 ml/2.5 × 10^6^ cells) of a methanol:MTBE:chloroform (MMC) mixture (40/30/30, v/v/v), containing 10 μg/100 mL of the antioxidant 2,6-di-t-butyl-p-hydroxytoluene (BHT). Subsequently, samples were vortexed, shaken (950 RPM; 30 min) and centrifuged at 8000 *rpm* for 10 min. A 2 μL aliquot of each sample was injected into a LC-MS system, consisting of a binary pump, a thermostated autosampler, a column compartment (Dionex UltiMate 3000 series; Thermo Fisher Scientific, Waltham, MA USA) and a Thermo Q-exactive mass spectrometer (Thermo Fisher Scientific, Waltham, MA USA). Liquid chromatography separation was performed at 45 °C using a Kinetex F5 reverse-phase column (Phenomenex inc.), flow rate of 0.65 ml/min. The mobile phases consisted of 5 mM ammonium formate and 0.1% formic acid in water (solvent A), and of 5 mM ammonium formate and 0.1% formic acid in isopropanol (solvent B). A gradient elution with 2 min run was used for lipid separation with the following steps: (a) starting solution, 20% solvent B; (b) 3 min, 40% solvent B; (c) 16 min, 60% solvent B; (c) 16.5 min, 70% solvent B; (d) 24 min, 74% solvent B; (e) 28 min, 95% solvent B. All solvents were purchased from Sigma-Aldrich and Biosolve (Dieuze, FR). In the first step, mass spectrometry analysis was performed with a positive/negative ion switching method in the Full MS scan mode. The Lipostar software (Molecular Discovery Ltd, UK) [38] was used to perform a pre-identification of potential lipid species on the basis of the m/z and retention time values, to generate *Inclusion Lists* aimed at obtaining MS/MS spectra. Thus, a reduced number of samples automatically selected by Lipostar to assure the coverage of the entire *Inclusion List* was analyzed again in DDA (Data-Dependent-Acquisition) mode using the *Inclusion List*. The workflow established for these experiments was followed by the identification process for each compound involving exact mass matching, retention time and MS/MS fragmentation. Automatically generated data were visually inspected, and only high-confidence data were ultimately selected for statistical analysis. The Statistical analysis module available in Lipostar was used to perform Principal Component Analysis (PCA) of the treated data by applying Pareto scaling.

### PCR studies

Total RNA was extracted with TRI Reagent^™^ and reverse-transcribed with a High-Capacity cDNA Reverse Transcription Kit, and the expression of selected genes was measured with TaqMan^™^ Gene Expression Assays and TaqMan^™^ Universal Master Mix II (Thermo fisher Scientific), according to the protocol provided by the manufacturer (polymerase activation: 95 °C, 10 min; PCR cycles: denaturation at 95 °C, 15 s; annealing at 60 °C, 1 min; 40 cycles), using a 7300 Real Time PCR System (Applied Biosystems). The amplification values of the 18 S RNA were used for the normalization of the data. The following Taqman assays were used for real-time PCR (Applied Biosystems): CRLS1 (Hs00219512_m1); TAZ (Hs00794094_m1); LCLAT1 (Hs00699427_m1); LPGAT1 (Hs00895487_m1); PGS1 (Hs00922697_m1); LPCAT1 (Hs00227357_m1).

### Mitochondrial studies

To define the functional activity and the mass of mitochondria, we stained cells with the Mito-Tracker Deep RED FM (Invitrogen) and Mito-Tracker Green FM kits (Invitrogen) contemporaneously, using the protocols provided by the manufacturer. Briefly, ~1 × 10^6^ cells for each experimental point were collected by centrifugation at the end of the treatment. Cells were resuspended in freshly prepared growth medium containing 2% formalin. After fixation, cells were rinsed in PBS (phosphate Buffer Saline) containing 1% BSA (Bovine Serum Albumin) several times before resuspension in the same buffer. Resuspended cells were subjected to quantitative analysis, using a fluorescence activated cell sorter (FACS, Becton and Dickinson). For the determination of red fluorescence the excitation and the emission wavelengths were 644 nm and 655 nm, respectively. For the determination of red fluorescence the excitation and the emission wavelengths were 490 nm and 516 nm, respectively. Mito-Tracker Deep Red FM is sensitive to the mitochondrial transmembrane potential, while the Mito-Tracker Green FM only to mitochondrial mass. Thus, the levels of red fluorescence provide a measure of the functional activity of mitochondria. By converse the level of green fluorescence provide a measure of the intracellular volume or mass of mitochondria. Calculation of the red/green fluorescence ratio normalizes the mitochondrial activity to the number of mitochondria [49].

To define the total mass of mitochondria, we also determined citrate synthase enzymatic activity in whole-cell extracts using a standard methodology described by Spinazzi et al. [50]. In fact citrate synthase is commonly employed as a quantitative enzyme marker for the presence of intact mitochondria.

The mitochondrial fractions of *NB4* and *NB4.306* cells as well as derived cellular populations (*shCTRL* and *shATG5*) were isolated from 4 × 10^7^ cells/experimental group with the use of a commercially available kit (Mitochondria Isolation Kit for Cultured Cells, Thermo Fisher Cat. No. 89874). Cell lysis was performed with 30 cycles of Dounce homogenization. Mitochondrial Complex-I, Complex-III, and Complex-IV enzymatic activities were measured in isolated mitochondria according to a described method with minor modifications [50]. Briefly, all the reactions were performed in 0.1 ml with a 96-well microtiter plate. We used 1–2 µg of mitochondrial protein-extracts/reaction and the absorbance was monitored with kinetic cycles (every 20 s for 10 min) in a TECAN Infinite M200 instrument, in the presence and absence of selective Complex-I, Complex-III, or Complex-IV inhibitors. The assays were performed with 4–6 replicates/experimental group, and the experiments were repeated with at least three biologically independent preparations. The results were normalized for the levels of citrate synthase activity determined in the mitochondrial fraction [50].

### FACS analyses and NBT-reductase activity

FACS analyses of CD11b, CD11c, and CD33 markers were performed according to standard procedures, which were used in various other previous studies performed with *NB4* and *NB4.306* cells [13, 17]. Specific phycoerythrin-conjugated monoclonal anti-CD11b, anti-CD11c, and anti-CD33 antibodies and relative negative controls were purchased from Beckton-Dickinson, Mountain View, CA. The ability of cells to reduce nitroblue-tetrazolium (*NBT*-reductase activity) was evaluated spectrophotometrically according to the method of Pick et al. [51], as described [13].

### Western blot analyses

Cell lysates collected in RIPA buffer (supplemented with 1 mM PMSF, 1× protease inhibitor) were separated by SDS–PAGE, transferred to a PVDF membrane (Immobilon-P, Merck Millipore Ltd), and incubated with primary antibodies (1:1000) at 4 °C overnight. After subsequent staining with HRP-conjugated secondary antibodies (1:10000 for anti-rabbit and 1:3000 for anti-mouse antibodies; 4 °C; overnight), the signal was developed using the ECL Star kit (Euroclone SpA, according to the instruction of the manufacturer using the BioRad Chemidoc^TM^ imaging system (Image Lab^TM^ touch software, BioRad). β2-actin was used as the loading control. Western blot experiments were performed with anti-ATG5, anti-PU.1, anti-Beclin1, anti-IRF1 (D5F5U; 9G7; D40C5; D5E4, Cell Signaling Technology), anti-LC3I/LCRII (MBL-PM036, MBL International), anti-CRLS1 (PA5–25338, Invitrogen), anti-LPCAT1, anti-PGS1 (PA5–26318; PA5–43247, Invitrogen) and anti-β2 actin (SC-47778, Santa Cruz) antibodies.

### Statistical Analyses

Differences between groups in the various experiments presented were determined following unpaired Student’s *t* tests, as detailed in the various Legends to Figures. The studies performed did not require power calculations and determination of sample sizes and we used a minimum of three independent cultures of cells to generate the results presented. Indeed, the type of cell-culture experiments, reported in the manuscript are characterized by low biological variability and strong biological signals. In our experimental conditions, three replicates/experimental group are sufficient to determine an effect size = 3.6 with a 5% statistical significance, a 90% power, and an allocation ratio of 1:1 (these calculations were performed with the G*Power software, version 3.1.9.2).

## Supplementary information

Reproducibility checklist

Supplementary Information

Supplementary Table S1

Supplementary Table S2

Supplementary Table S3

Supplementary Table S4

Supplementary Table S5

## Data Availability

The *RNA-seq* data were deposited in the EMBL-EBI Arrayexpress database (Accession No: E-MTAB-10267). All the other data generated or analyzed during this study are included in this published article and its supplementary information files.
