# Supplementary material for: Role of cardiolipins, mitochondria, and autophagy in the differentiation process activated by all-trans retinoic acid in acute promyelocytic leukemia
Source: Cell Death Dis. 2022 Jan 10;13(1):30. doi: 10.1038/s41419-021-04476-z (PMC8748438; doi:10.1038/s41419-021-04476-z)
Supplement: Supplementary file 2 — Supplementary Information [file 41419_2021_4476_MOESM2_ESM.pdf]

SUPPLEMENTARY INFORMATION

Title: Role of cardiolipins, mitochondria and autophagy in the differentiation process activated by all-trans retinoic acid in acute promyelocytic leukemia

Maurizio Gianni'[1], Laura Goracci[2], Anna Schlaefli[3], Alessandra Di Veroli[2], Mami Kurosaki[1], Luca Guarrera[1], Marco Bolis[1,4,5], Marika Foglia[1], Monica Lupi[6], Mario P. Tschan[3], Gabriele Cruciani[2], Mineko Terao[1] and Enrico Garattini[1]

[1] Laboratory of Molecular Biology, Istituto di Ricerche Farmacologiche Mario Negri IRCCS, via Mario Negri 2, 20156 Milano, Italy.

[2] Department of Chemistry, Biology and Biotechnology, University of Perugia, via Elce di Sotto 8, 06123 Perugia, Italy

[3] Institute of Pathology, University of Bern, Murtenstrasse 31, CH-3008 Bern, Switzerland.

[4] Functional Cancer Genomics Laboratory, Institute of Oncology Research, USI, University of Southern Switzerland, 6500, Bellinzona, Switzerland.

[5] Bioinformatics Core Unit Institute of Oncology Research, Swiss Institute of Bioinformatics, 1000, Lausanne, Switzerland.

[6] Department of Oncology, Istituto di Ricerche Farmacologiche "Mario Negri" IRCCS, via Mario Negri 2, 20156 Milano, Italy.

Legends to Supplementary Tables and Figures page: 2-6

Supplementary Figures S1-S6 page: 7-12

LEGENDS TO SUPPLEMENTARY TABLES AND FIGURES

Supplementary Table S1 *Lipidomic data obtained in retinoid-sensitive NB4 and retinoid-resistant NB4.306 cells* The amounts of the indicated lipids were determined with the Lipostar high-throughput software mentioned in the MATERIALS AND METHODS section of the manuscript. The data were obtained from 3 independent cultures of logarithmically growing *NB4* and *NB4.306* cells which were exposed to vehicle (DMSO) or ATRA (1 μM) for 6, 24 and 48 hours as indicated. The values are expressed in total counts and they were obtained from the mass spectrometry results. The Mean and Standard Deviation (SD) values of the triplicate samples are marked in red and green, respectively.

Supplementary Table S2 *Effects of ATRA on the gene expression profiles of NB4 cells* The effects exerted by ATRA on the transcriptomic profiles of parental *NB4* cells were evaluated using the *RNA-seq* data generated from 3 independent cultures of cells exposed to vehicle (DMSO) or ATRA (1 μM) for 48 hours. The first sheet (Total gene expression data) shows the expression levels of all the genes identified in our experimental conditions. The second sheet shows the result of a pathway enrichment analysis performed with the *RNA-seq* data contained in sheet 1, using the Gene Ontology (GO) annotations.

Supplementary Table S3 *Effects of ATRA on the expression of genes coding for mitochondrial proteins in NB4 cells* The effects exerted by ATRA on the expression of genes coding for mitochondrial proteins in parental *NB4* cells were evaluated using the *RNA-seq* data generated from 3 independent cultures of cells exposed to vehicle (DMSO) or ATRA (1 μM) for 48 hours. The list of the mitochondrial genes considered is based on the annotations available in the following Gene Ontology datasets: mitochondrial small ribosomal subunit (GO:0005763); mitochondrial outer membrane translocase complex (GO:0005742); mitochondrial outer membrane (GO:0005741);

mitochondrial respiratory chain complex IV (GO:0005751); mitochondrial inner membrane presequence translocase complex (GO:0005744); mitochondrial matrix (GO:0005759); mitochondrial large ribosomal subunit (GO:0005762); mitochondrial proton-transporting ATP synthase complex (GO:0005753); integral component of mitochondrial membrane (GO:0032592); intrinsic component of mitochondrial inner membrane (GO:0031304); mitochondrial respiratory chain complex III (GO:0005750); integral component of mitochondrial outer membrane (GO:0031307); integral component of mitochondrial inner membrane (GO:0031305); mitochondrial proton-transporting ATP synthase complex, coupling factor F(o) (GO:0000276); intrinsic component of mitochondrial outer membrane (GO:0031306); mitochondrial envelope (GO:0005740); mitochondrial inner membrane (GO:0005743); mitochondrial respiratory chain complex I (GO:0005747); mitochondrion (GO:0005739); mitochondrial intermembrane space (GO:0005758). The mitochondrial genes whose expression is significantly down-regulated and up-regulated by ATRA are marked in blue and red, respectively. The genes whose expression is left unaltered by ATRA are marked in black. The set of 13 genes derived from mitochondrial DNA (MT-) is shown as a separate group.

Supplementary Table S4 *Lipidomic data obtained in NB4-shCTRL and NB4-shATG5 cells* The amounts of the indicated lipids were determined with the Lipostar high-throughput software mentioned in the MATERIALS AND METHODS section of the manuscript. The data were obtained from 3 independent cultures of logarithmically growing *NB4-shCTRL* and *NB4-shATG5* cells which were exposed to vehicle (DMSO) or ATRA (1μM) for 24 and 48 hours as indicated. The values are expressed in total counts and they were obtained from the mass spectrometry results. The Mean and Standard Deviation (SD) values of the triplicate samples are marked in red and green, respectively.

Supplementary Table S5 *Effects of ATRA on the gene expression profiles of NB4-shCTRL and NB4-shATG5 cells* The effects exerted by ATRA on the transcriptomic profiles of *NB4-shCTRL and NB4-shATG5* cells were evaluated using the *RNA-seq* data generated from 3 independent cultures of cells exposed to vehicle (DMSO) or ATRA (1 μM) for 48 hours. The table shows the expression levels of all the genes identified in our experimental conditions.

Supplementary Figure S1 *Effects of ATRA on the lipidomic profiles of retinoid-sensitive NB4 and retinoid-resistant NB4.306 cells* Three independent cultures of logarithmically growing *NB4* and *NB4.306* cells were treated with vehicle (DMSO) or ATRA (1 μM) for 6, 24 and 48 hours. (**A**) The panels illustrate the bidimensional principal component (PC) analysis of the lipidomic data obtained in our experimental conditions. (**B**) The column diagram shows the types and number of lipid entities determined in our experimental conditions. The values in parenthesis indicates the number of different molecular entities identified for each subclass of lipids. Cardiolipins (*CLs*) are marked in red, as they represent the major object of the study.

Supplementary Figure S2 *Effects of ATRA on specific subclasses of lipids in retinoid-sensitive NB4 and retinoid-resistant NB4.306 cells* Three independent cultures of logarithmically growing *NB4* and *NB4.306* cells were treated with vehicle (DMSO) or ATRA (1 μM) for 6, 24 and 48 hours. The Figure shows that ATRA does not cause significant alterations in the levels of the indicated subclasses of lipids in either *NB4* or *NB4.306* cells. The number of molecular species identified for each lipid subclass are indicated in parenthesis. Each box-plot represents the Median values ± SD of the various lipid species determined in the 3 independent cultures treated with vehicle or ATRA as indicated.

Supplementary Figure S3 *Effects of ethanolamine on citrate synthase activity in NB4 cells exposed to ATRA* Three independent cultures of retinoid-sensitive *NB4* cells were pre-treated with 20 and 50

μM ethanolamine (ET) for 12 hours. Subsequently cells were treated with vehicle (DMSO) or ATRA (1 μM) for another 48 hours. The concentration of ET used (20 μM; 50 μM) is indicated by the numbers shown under the diagram. Citrate synthase activity was measured in cell homogenates. Each value is the Mean±S.D. of the 3 independent cultures.

Supplementary Figure S4 *Ethanolamine and ATRA effects on PML-RARα and RARα proteins in NB4 cells* Three independent cultures of logarithmically growing *NB4* cells were pre-treated with the indicated concentrations of ethanolamine (ET) or vehicle (DMSO) for 24 hours. Subsequently cells were exposed to ATRA (1μM) or DMSO for a further 24 hours. Pooled cell homogenates obtained from the three independent cultures were electrophoresed and subjected to Western blot analysis with anti-RARα antibodies to determine the levels of PML-RARα and RARα, as indicated. The levels of $\beta_2$actin indicate that the same amount of proteins was loaded in each lane of the gel. The molecular weights (MW) of the indicated proteins are shown on the right.

Supplementary Figure S5 *Effects of ATRA on the lipidomic profiles of NB4-shCTRL and NB4-shATG5 cells* Three independent cultures of logarithmically growing *NB4-shCTRL* and *NB4-shATG5* cells were treated with vehicle (DMSO) or ATRA (1μM) for 24 and 48 hours. (**A**) The column diagram shows the types and number of lipid species determined in our experimental conditions. The values in parenthesis indicates the number of different molecular entities identified for each subclass of lipids. The lipid subclasses marked with a black arrow above the corresponding columns are the same lipid subclasses identified in parental *NB4* and *NB4.306* cells (see Suppl.Fig.S1A). Cardiolipins (*CLs*) are marked in red, as they represent the major object of the study. (**B**) The panels illustrate the bidimensional principal component (PC) analysis of the lipidomic data obtained in our experimental conditions.

Supplementary Figure S6 *Effects of ATRA on specific subclasses of lipids in NB4-shCTRL and NB4-shATG5 cells* Three independent cultures of logarithmically growing *NB4-shCTRL* and *NB4-shATG5* cells were treated with vehicle (DMSO) or ATRA (1μM) for 24 and 48 hours. The Figure shows the effects of ATRA on the levels of the indicated lipid subclasses in *NB4-shCTRL* and *NB4-shATG5* cells. The number of molecular entities identified for each lipid subclass are indicated by the number in parenthesis. Each box-plot represents the Median values $\pm$ SD of the various lipid entities determined in the 3 independent cultures treated with vehicle or ATRA as indicated. *Significantly different relative to the corresponding vehicle treated control ($p<0.05$, Student's t-test). **Significantly different relative to the corresponding vehicle treated control ($p<0.01$, Student's t-test). *diacylglycerophosphocholines* and *monoacylglycerophosphocholines* are marked with a red arrow, as they are among the lipid groups which are significantly down-regulated by ATRA in parental NB4 cells (see Fig.1).

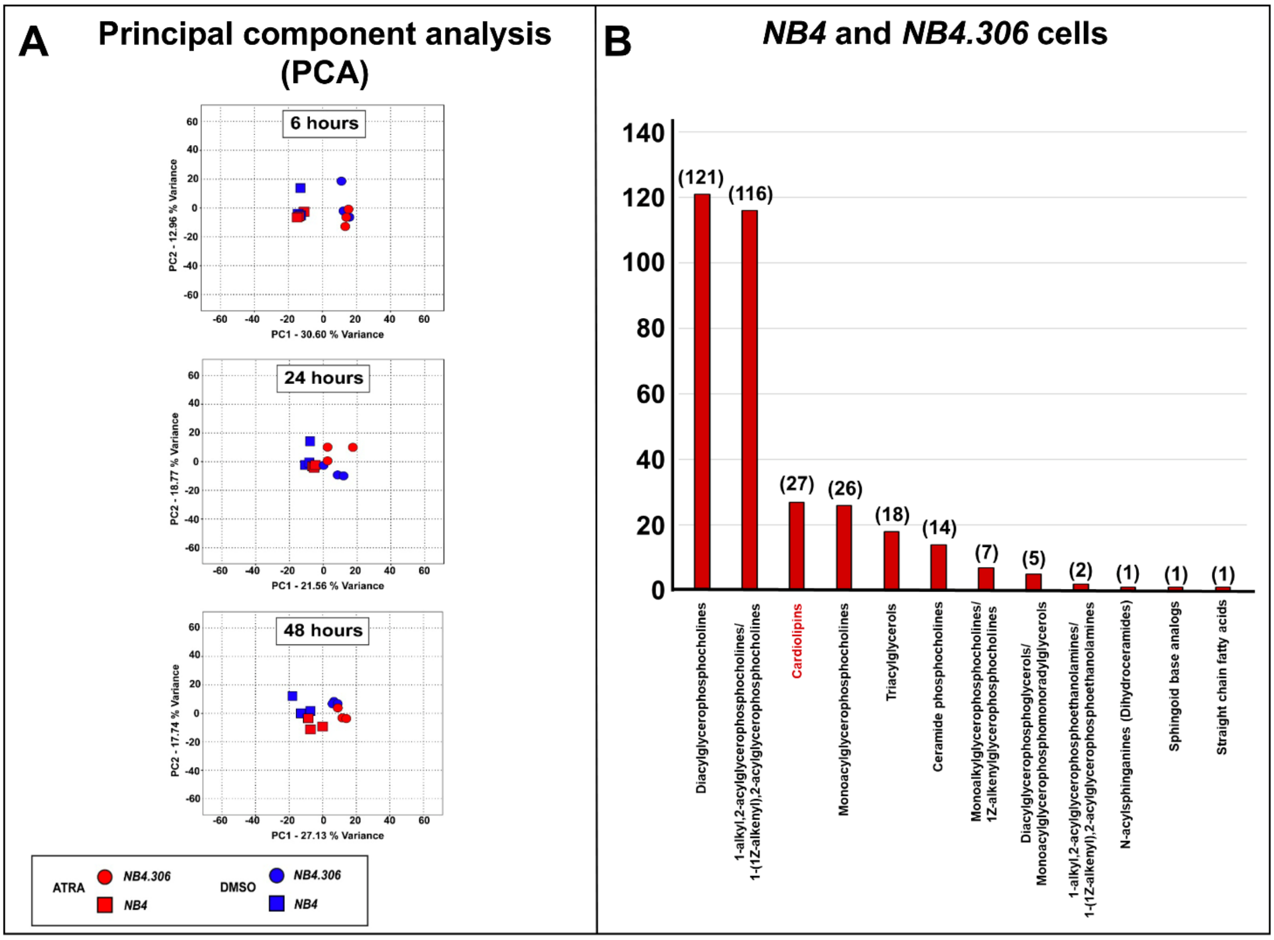

Supplementary Figure S1 *Effects of ATRA on the lipidomic profiles of retinoid-sensitive NB4 and retinoid-resistant NB4.306 cells*

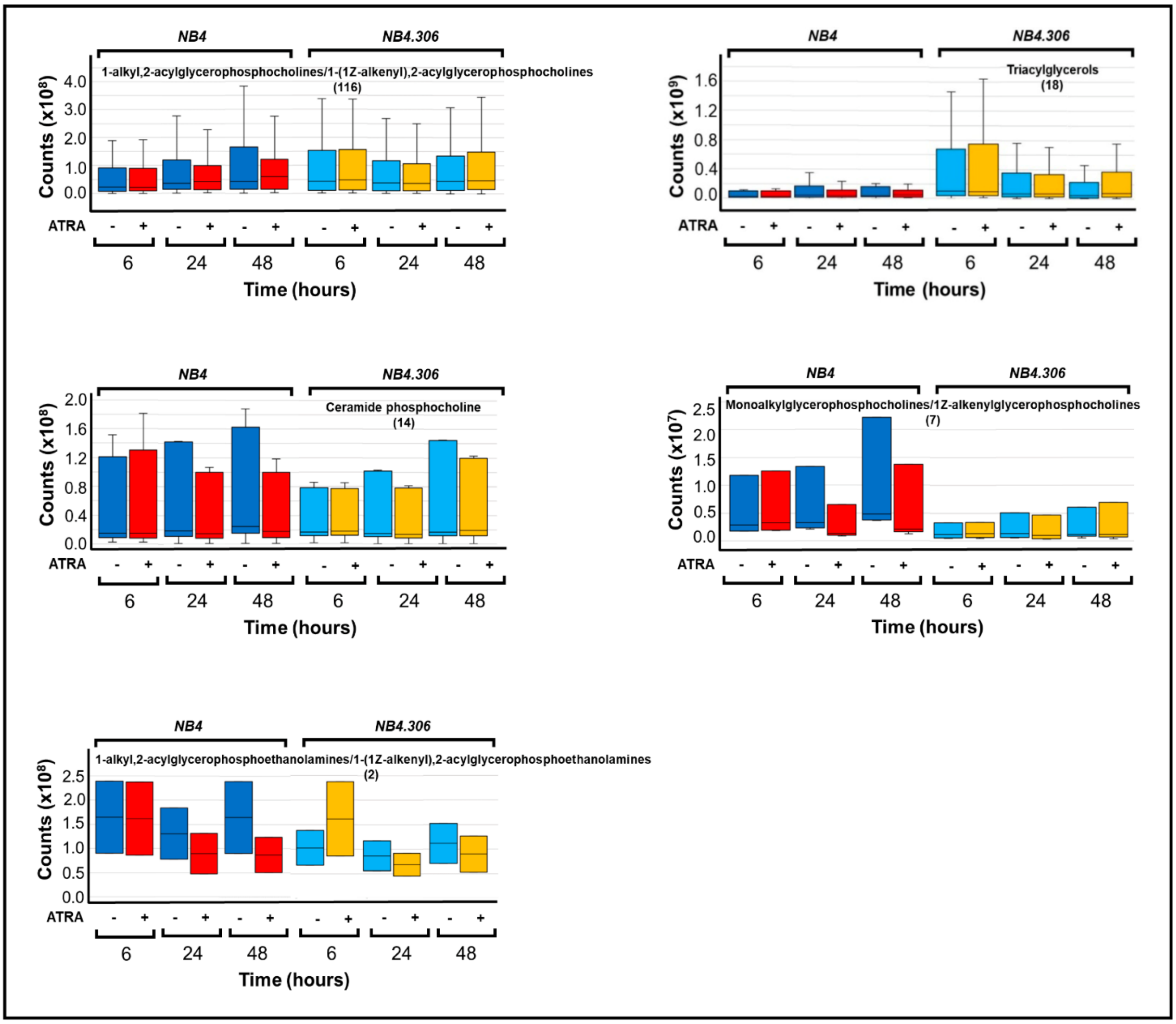

Supplementary Figure S2 *Effects of ATRA on specific subclasses of lipids in retinoid-sensitive NB4 and retinoid-resistant NB4.306 cells*

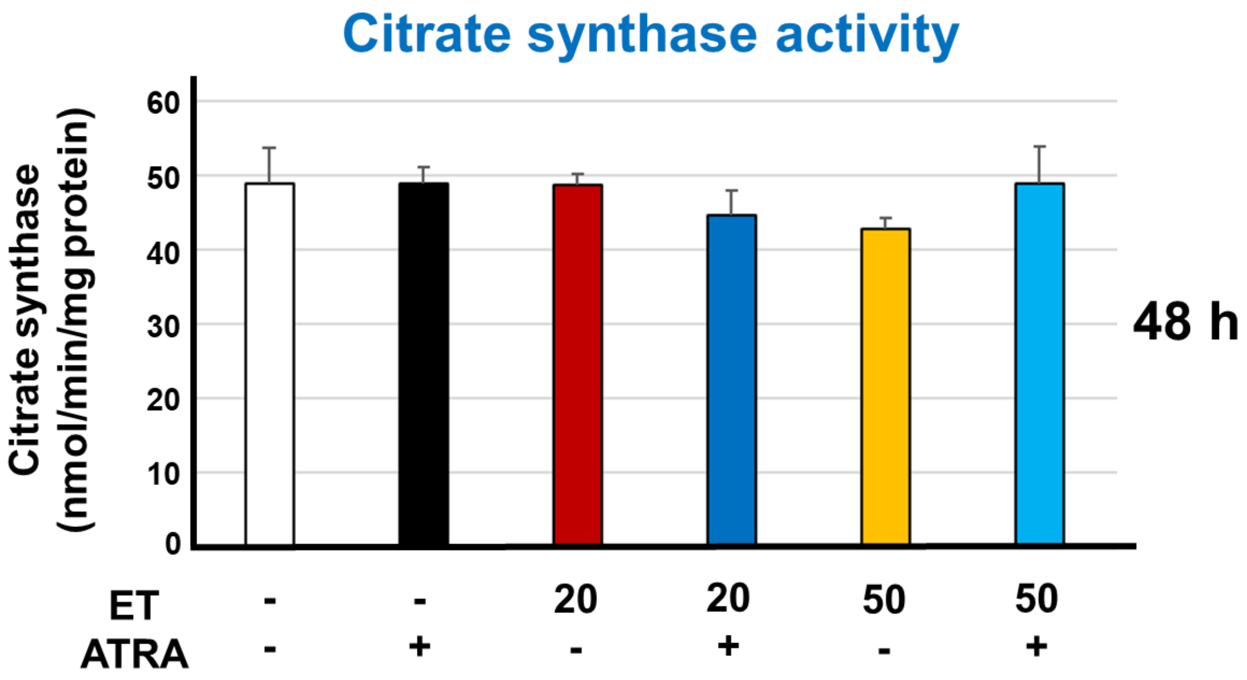

Supplementary Figure S3 *Effects of ethanolamine on citrate synthase activity in NB4 cells exposed to ATRA*

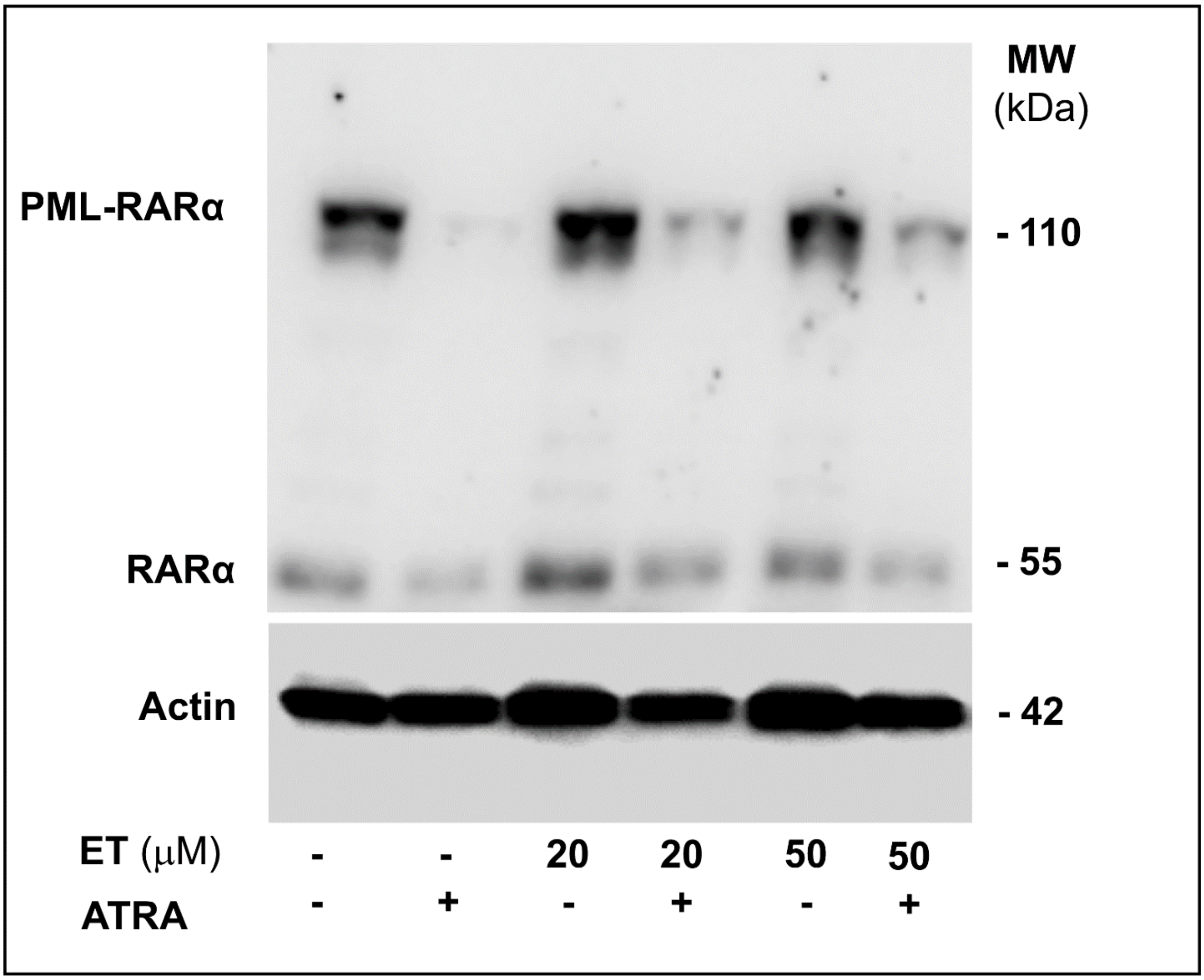

Supplementary Figure S4 *Ethanolamine and ATRA effects on PML-RARα and RARα proteins in NB4 cells*

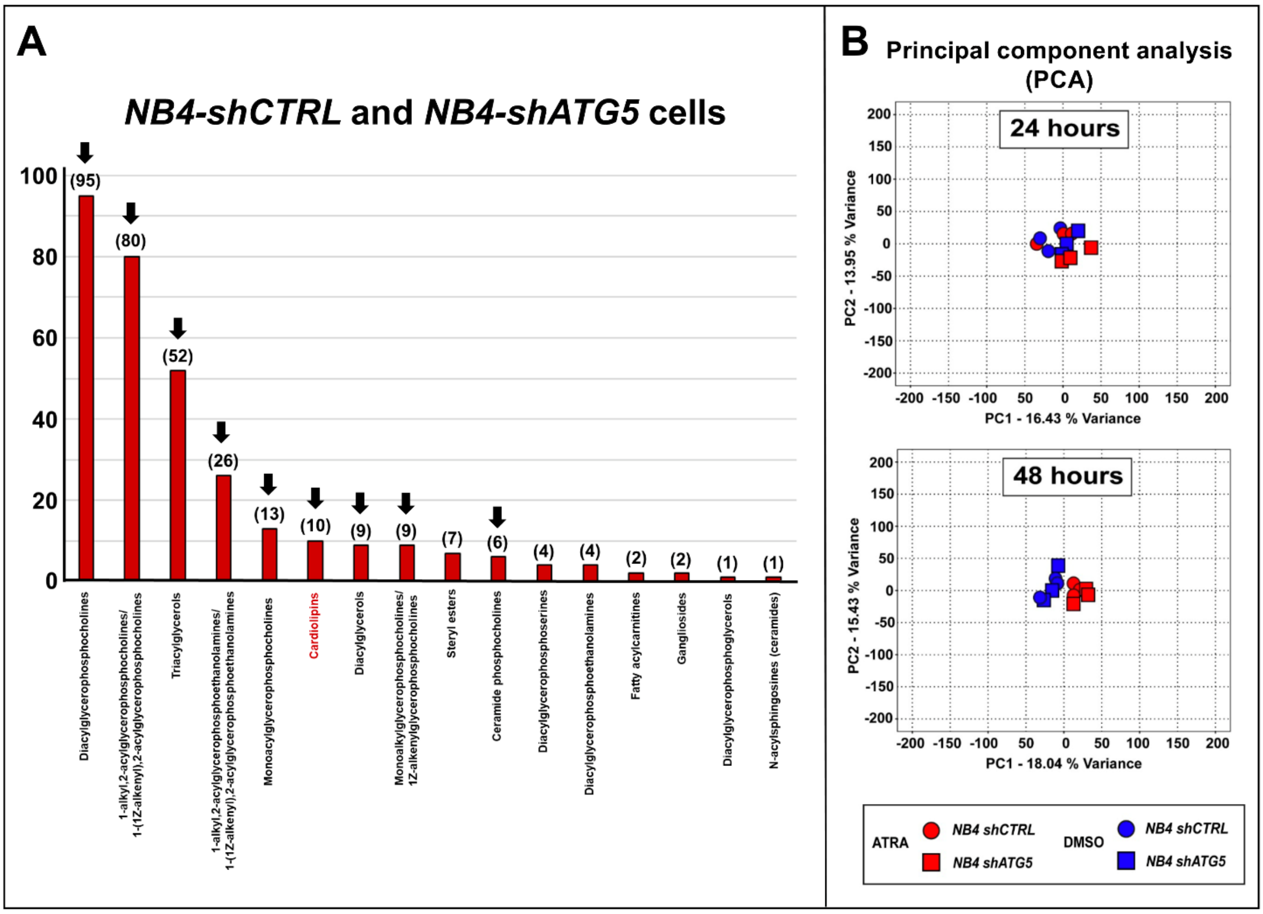

Supplementary Figure S5 *Effects of ATRA on the lipidomic profiles of NB4-shCTRL and NB4-shATG5 cells*

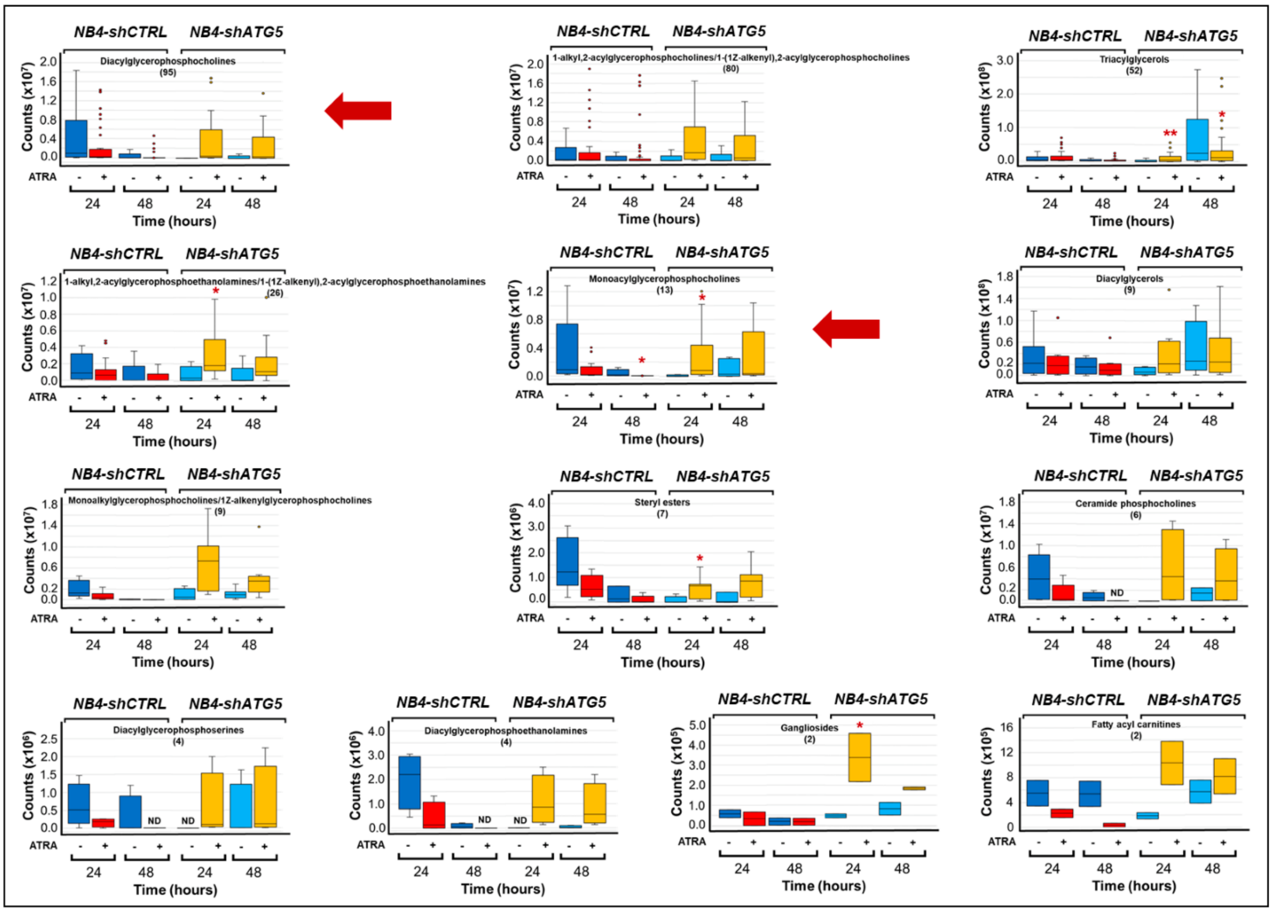

Supplementary Figure S6 *Effects of ATRA on specific subclasses of lipids in NB4-shCTRL and NB4-shATG5 cells*
